# Modelling clinical DNA fragmentation in the development of universal PCR-based assays for bisulfite-converted, formalin-fixed and cell-free DNA sample analysis

**DOI:** 10.1038/s41598-022-18196-7

**Published:** 2022-09-26

**Authors:** Andrew D. Johnston, Jennifer Lu, Darren Korbie, Matt Trau

**Affiliations:** 1 Centre for Personalized NanoMedicine, The University of Queensland, St Lucia, QLD 4072 Australia; 2 Australian Institute for Bioengineering and Nanotechnology, The University of Queensland, St Lucia, QLD 4072 Australia; 3 Molecular Diagnostics Solutions, CSIRO Health and Biosecurity, Westmead, NSW Australia; 4 School of Chemistry and Molecular Biosciences, The University of Queensland, St Lucia, QLD 4072 Australia

**Keywords:** Cancer, Genetics, Epigenetics, Genomics

## Abstract

In fragmented DNA, PCR-based methods quantify the number of intact regions at a specific amplicon length. However, the relationship between the population of DNA fragments within a sample and the likelihood they will amplify has not been fully described. To address this, we have derived a mathematical equation that relates the distribution profile of a stochastically fragmented DNA sample to the probability that a DNA fragment within that sample can be amplified by any PCR assay of arbitrary length. Two panels of multiplex PCR assays for quantifying fragmented DNA were then developed: a four-plex panel that can be applied to any human DNA sample and used to estimate the percentage of regions that are intact at any length; and a two-plex panel optimized for quantifying circulating cell-free DNA (cfDNA). For these assays, regions of the human genome least affected by copy number aberration were identified and selected; within these copy-neutral regions, each PCR assay was designed to amplify both genomic and bisulfite-converted DNA; and all assays were validated for use in both conventional qPCR and droplet-digital PCR. Finally, using the cfDNA-optimized assays we find evidence of universally conserved nucleosome positioning among individuals.

## Introduction

Three DNA sample types commonly encountered in cancer research are bisulfite-converted DNA, DNA extracted from formalin fixed paraffin embedded (FFPE) tissue, and circulating cell-free DNA (cfDNA). One feature these sample types have in common is their high degrees of fragmentation, which makes them difficult to quantify accurately^1,2^. Working with, and designing PCR assays for, each of these sample types poses its own set of unique challenges. Furthermore, bisulfite conversion can be applied to both FFPE and cfDNA samples, further compounding these challenges.

Bisulfite conversion involves treating DNA with bisulfite (HSO_3_^−^), which converts all unmethylated cytosines to uracil (sequenced as thymine) while leaving methylated cytosines unchanged. When a bisulfite region is sequenced the methylated and unmethylated cytosines can be discerned by reference to the known unconverted genomic sequence. The harsh chemical treatment of the bisulfite conversion process results in substantial DNA fragmentation. As for FFPE, upon extraction tumour tissues must be preserved and stored to allow for subsequent testing and use in future research. Such samples are often stored as FFPE specimens, which, over time, leads to substantial DNA degradation^3,4^. Furthermore, formalin fixation causes the formation of molecular crosslinks (i.e., covalent bonds between DNA and proteins)^5,6^. The process of extracting DNA from FFPE blocks and reversing crosslinking leads to further fragmentation^7^. When designing PCR assays it is therefore important to consider the effects that fragmentation and crosslinking might have on primer binding and subsequent amplification.

Finally, cfDNA is a source of DNA with particularly high levels of fragmentation that is becoming increasingly clinically relevant. cfDNA is found in the blood of healthy individuals due to the regular apoptosis of white blood cells, among other cell types from healthy tissues^8,9^. However, cancer can also contribute circulating tumour DNA (ctDNA) to this mix^10^. Unlike bisulfite-converted and FFPE-derived DNA, cfDNA deviates substantially from a stochastic fragmentation model. Whole-genome sequencing of cfDNA from both healthy donors and cancer patients has revealed non-random wave-like coverage patterns that instead align with a nucleosome occupancy model of DNA fragmentation^11–14^.

In DNA that has been randomly fragmented from processes such as ultra-focused sonication, bisulfite conversion, or storage in, and extraction from, FFPE blocks, the number of PCR-amplifiable copies of DNA is a function of both the length of the target region and the degree of fragmentation of the sample. A corollary of this model of random fragmentation is that while the total number of amplifiable DNA copies in the sample will decrease as the length of the region is increased, fragments of identical length should represent a population that contains all portions of the genome equally, regardless of where in the genome those fragments originated. Amplicons of the same length should, therefore, measure the same number of copies in quantitative PCR, and whole-genome sequencing should produce uniform coverage in randomly fragmented samples.

In this study we present a fundamental equation describing stochastic DNA fragmentation that can be applied to fragment size distribution data to determine the proportion of a DNA region that remains intact in a randomly fragmented sample. Moreover, we demonstrate that quantitation by two PCR assays of different lengths can be used to estimate intact proportion, average fragment length, and number of genome copies (i.e., the sum of intact and broken target regions) in a randomly fragmented sample, based on the strong correlation of our model with experimental results. To address the need for a technique that accurately quantifies both the concentration and fragmentation of cancer-derived DNA, we design these PCR assays as a universal quantification 4-plex that works for both quantitative real-time PCR (qPCR) and droplet digital PCR (ddPCR). This 4-plex includes amplicons at two different sizes, enabling a ratiometric measure of DNA fragmentation, and these amplicons target unique regions rarely affected by copy number aberrations in cancer, enabling accurate quantification of samples derived from cancer patients. Furthermore, each assay targets a separate chromosome, thus providing internal copy number controls for validation and quality control. To further enhance quantification under a variety of experimental and clinical conditions we use a method first described by Lofton-Day (2008)^15^ of targeting cytosine-free priming sites, allowing the amplification of both genomic and bisulfite-converted human DNA. We use these assays to compare quantification of fragmented DNA by PCR-based methods to other techniques, as well as the recovery rates and degree of fragmentation of three commonly used commercial bisulfite conversion kits to validate the performance and utility of this multiplexed universal quantification assay.

Finally, we design two additional PCR-based assays for accurate quantification of cfDNA samples. Snyder et al. (2016)^11^ recently used deep sequencing of cfDNA samples to map nucleosome positions based on local peaks in nucleosome-mediated cfDNA protection. We use these data to target one nucleosome peak with relatively weak protection and one peak with relatively strong protection. As with the 4-plex quantification assays, we target cytosine-free regions rarely affected by copy number aberrations. Furthermore, we design 8 assays flanking these two protection peaks to explore the effects of amplicon length and distance from these peaks on cfDNA concentration.

## Results

### Modelling random DNA fragmentation

**Figure 1:**
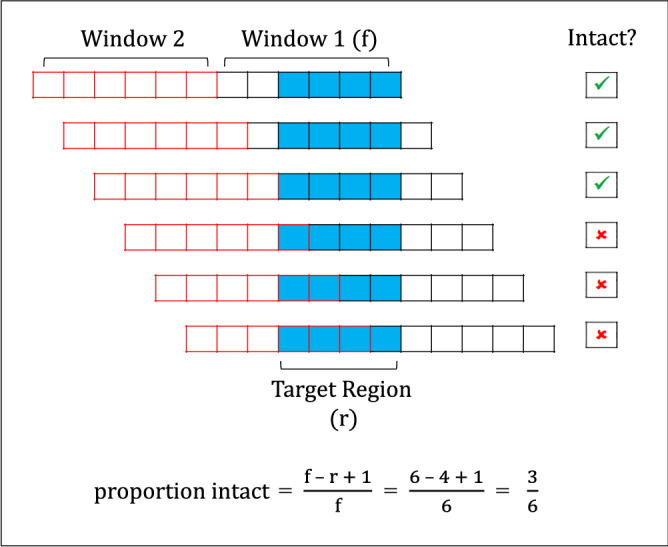
Diagram depicting example calculation of the proportion of intact copies of a target region (4 bp) given a single specified fragment length (6 bp). This calculation can be viewed as the probability that a region will not be cleaved at any point along its length if a genome were broken into equal length fragments. The fragment-sized Window 1 sliding across this region depicts all possible fragmentation states for this region. The intact proportion is calculated as the number of states where the region remains entirely within the fragment window over the total number of possible fragmentation states. Window 2 demonstrates that all possible states are represented at the point before the region fully exits Window 1, as these states are then repeated in this adjacent window.

To begin our study, we required a model that would accurately reflect the properties of a stochastically fragmented DNA sample. The odds that a region targeted by a PCR assay will be interrupted by a DNA breakage in randomly fragmented DNA depend on the length of the region and the size of the fragments. These odds are effectively determined by establishing two adjacent fragment-sized sliding windows (wherein the end of one fragment is the start of another) and calculating the number of times a region is fully within the first fragment window, compared to the number of times the region is situated within both windows (Fig. 1).

This model is represented in Eq. (1), which determines the probability that a region of DNA will remain unbroken for a given fragment length:

1 $${\text{proportion}}\;{\text{intact}} = \frac{{{\text{f }}{-}{\text{ r }} + { }1}}{{\text{f}}},$$

where r is the length of the DNA region and f is the length at which the DNA is fragmented. However, DNA samples do not fragment at a single length but rather as a distribution, and by incorporating size distribution profiles, which contain the concentration of DNA at each fragment length, the proportion of intact target regions within a fragmented DNA sample can be calculated, as detailed in Eq. (2):

2 $${\text{proportion}}\;{\text{intact }} = \frac{{\mathop \sum \nolimits_{{f = r{ }}}^{n} \frac{{{\text{f }}{-}{\text{ r }} + { }1}}{{\text{f }}}{ }C_{f} }}{{\mathop \sum \nolimits_{{f = m{ }}}^{n} C_{f} }},$$

where n is the length of the longest fragment within the sample, m is the length of the shortest fragment, and C_*f*_ is the concentration of each fragment length (i.e., pg/µl).

### Designing universal PCR assays for fragmented clinical cancer samples

We next sought to design qPCR and ddPCR assays that could be used to interrogate DNA fragmentation. A major focus of this assay design was to incorporate design elements that would enable the assays to be used on clinical cancer samples, as these samples are some of the most common types to undergo stochastic fragmentation. However, cancer samples are also prone to chromosomal amplifications and deletions within the genome^16–18^, and PCR assays that intersected with frequently amplified/deleted regions would result in inaccurate measures of concentration when these copy number aberrations (CNAs) occurred (i.e., the concentration of a region that is unique in the human reference genome is assumed to correspond to the overall number of genome copies within the measured sample). To control for this, we undertook an analysis to determine the regions of the human genome that were least affected by CNAs. CNA data that had been tested for statically significant gain or loss was retrieved from the Catalogue of Somatic Mutations in Cancer (COSMIC release v78)^19,20^. This data was filtered to exclude cell line samples and samples missing total copy number or minor allele values. Only 27 of the 10,637 samples remaining after this filtering were not derived from The Cancer Genome Atlas (TCGA) data^21^. We, therefore, opted to exclusively use these 10,610 TCGA samples to better ensure a dataset with experimental and analytical consistency in determining copy number changes (S1 Table).

**Figure 2:**
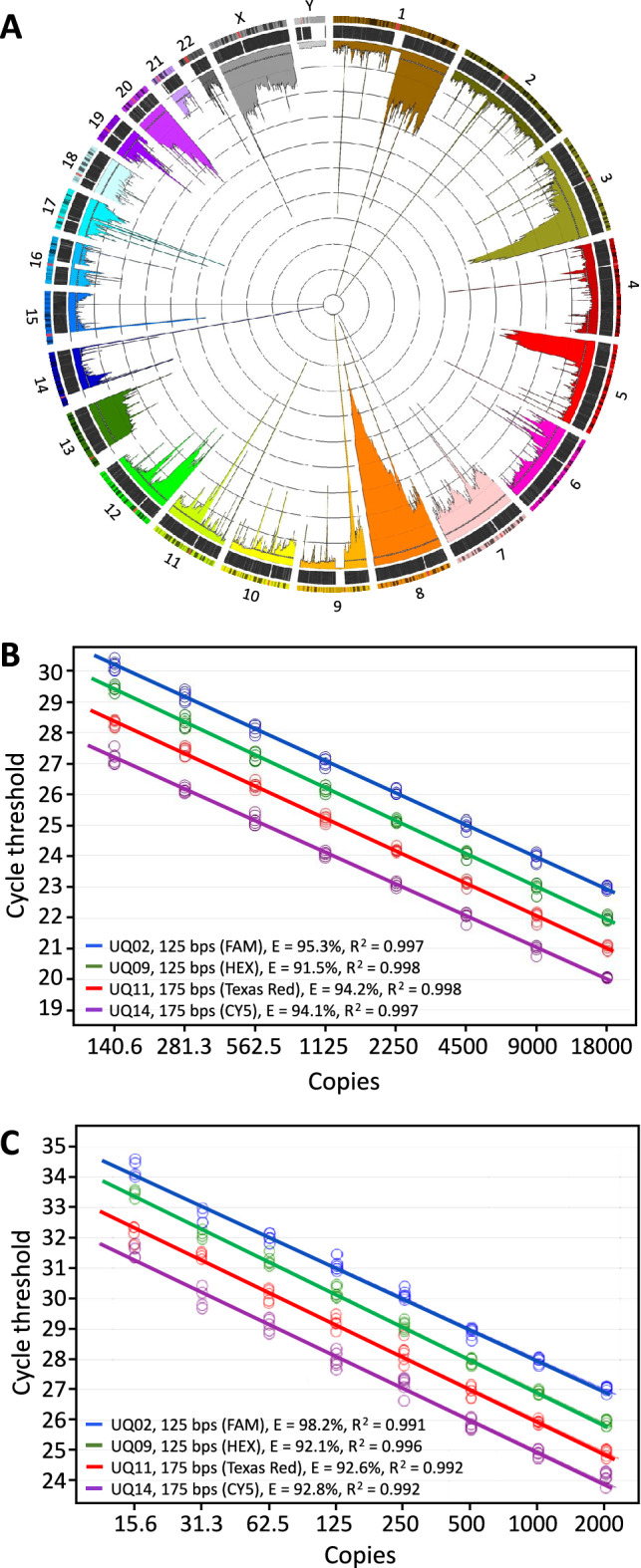
Design and performance of PCR assays against copy-neutral regions in the genome. (**A**) Circos plot depicting the percent of samples that undergo copy number aberration (CNA) in cancer. Chromosomes are shown in the outermost ring and include an overlay of cytogenetic Giemsa banding and centromeres marked with a red band. The second outermost ring shows the 946,615 Affymetrix Genome-Wide Human SNP Array 6.0 copy number probes used for the detection of CNAs by the TCGA. The final layer is a histogram displaying the number of samples that underwent statistically significant CNA (either loss or gain) within each region. Each grid line represents 1% of the 10,610 total samples. Universal assays were designed to target regions in the bottom 10th percentile of CNAs, excluding regions that are not covered by the Affymetrix CNV probes (e.g., centromeres). Less than 35 of the 10,610 samples (< 0.33%) have CNAs in these regions, represented on the histogram as a dotted white line (above which regions were excluded). (**B**, **C**) Standard curves estimating amplification efficiencies of universal quantitation assays in 4-plex qPCR on gDNA (**B**) and bisulfite-converted DNA (**C**). Curves are artificially offset for better visualisation. E = efficiency.

After filtering out regions that were not covered by Affymetrix copy number probes (e.g., centromeres) the only regions completely devoid of CNAs were telomeric and likely artefactual. Outside of telomeres the minimum CNA region contained 5 samples. To determine a reasonable threshold for low copy number variation that might provide us with enough region space to meet the requirements of our assay design, we calculated the number of samples with CNAs in commonly used copy number reference genes. We found that the “Human TaqMan Copy Number Reference Assays” targeting RNase P and TERT offered by Applied Biosystems had CNAs in 61 and 360 of the total 10,610 samples, respectively, and the well-established standard reference gene RPP30 had CNAs in 23 samples. Based on this we set a threshold at the bottom 10th percentile of regions, excluding those where greater than 34 samples had significant copy number variation (Fig. 2A). After applying this filter, we were left with 621 megabases across 858 non-contiguous regions on 22 chromosomes.

We next designed a single-tube 4-plex quantitative PCR assay targeting these CNA neutral regions, which included a variety of design considerations to maximize the utility of the assay and minimize confounding effects. First, each assay would target a separate chromosome to minimize inaccurate quantification due to the remote possibility that one of the chromosomes, or at least a large portion, may be affected by CNAs. Given the size and number of regions, the second design consideration was identifying assay regions that would be unaffected by bisulfite conversion treatment, since the bisulfite conversion process is used to examine DNA methylation and is a common application in cancer genomics but also leads to substantial sample fragmentation and loss. To address this design consideration the CNA neutral regions were further analysed to identify primer and probe regions that were cytosine-free and would, therefore, be unaffected by the bisulfite conversion process. Notably, use of the assays on bisulfite material requires an extra step in qPCR data analysis to correct for the fact that only one DNA strand is quantified, resulting in a positive shift of 1 cycle threshold when compared to the unconverted genomic DNA (gDNA) counterpart.

The third design criterion was to enable assessment of the degree of sample fragmentation using this 4-plex assay. To achieve this, two of the assays were designed to be 125 bp in length, and two were designed to be 175 bp long. By taking the ratio of concentrations for the long to short assays, a quantitative metric for sample fragmentation can be imputed for any sample.

Finally, we sought to establish the combination of fluorescent probe chemistries that would enable successful multiplexing quantitation using either standard qPCR or ddPCR. In qPCR four different probe fluorophores (FAM, HEX, Cy5 and Texas Red) were used, whereas ddPCR 4-plex was achieved using a method developed by Dobnik et al. (2016)^22^ that uses two FAM probes and two HEX probes and varies probe concentrations to alter the resulting levels of fluorescence amplitude, allowing for the detection of two targets per fluorescence channel (S1 and S2 Figs).

After all these design criteria were successfully implemented, we next undertook experiments to verify the amplification fidelity and efficiency of each of the four assays. The fidelity of the assays was established by performing standard PCR and qPCR on a variety of sample types (buffy coat DNA, cfDNA, and bisulfite-converted DNA) and analysing the PCR products by standard DNA gel electrophoresis to confirm that only a single PCR amplicon was produced in singleplex (S3 Fig), and that multiplex assays produced only two bands of the expected sizes (S4 and S5 Figs). Next, the amplification efficiencies of all assays were determined using LinRegPCR window-of-linearity analysis^23^, and standard titration curves; this was done for all four amplicons in both singleplex and multiplex configurations, for both genomic and bisulfite-converted DNA, using both fluorescent dye and PrimeTime qPCR probes in multiple fluorophore configurations (Fig. 2B,C, Table 1). Notably, all assays demonstrated > 90% amplification efficiency across all conditions, indicating robust performance. Primer and probe sequences can be found in S2 Table.

**Table 1:** Universal 4-plex qPCR amplification efficiencies.

Multiplex name	Amplicon size (bp)	Assay	Chr	Fluorophore	DNA	Efficiency			
						Standard curve		Window-of-linearity	
						%	R^2^	Median (%)	MAD
Singleplex	125	UQ02	2	SYTO 9	Genomic	93.1	0.997	94.5	3.6
					Bisulfite	96.7	0.997	102.0	1.6
Singleplex	125	UQ09	9	SYTO 9	Genomic	93.3	0.999	104.1	1.2
					Bisulfite	97.3	0.997	97.7	3.0
Singleplex	175	UQ14	14	SYTO 9	Genomic	91.3	0.997	96.9	1.2
					Bisulfite	90.3	0.993	112.9	1.1
Singleplex	175	UQ11	11	SYTO 9	Genomic	90.7	0.998	103.9	2.8
					Bisulfite	92.1	0.997	103.4	3.3
2-plex	125	UQ02	2	FAM	Genomic	92.3	0.999	91.6	4.8
					Bisulfite	92.0	0.993	95.9	1.3
	175	UQ14	14	HEX	Genomic	93.0	0.999	98.0	7.0
					bisulfite	94.3	0.994	93.6	3.8
2-plex	125	UQ09	9	FAM	Genomic	94.3	0.999	100.1	13.1
					Bisulfite	91.7	0.997	99.4	6.1
	175	UQ11	11	HEX	Genomic	93.8	0.999	92.7	8.0
					Bisulfite	94.0	0.995	87.9	8.8
4-plex	125	UQ02	2	FAM	Genomic	95.3	0.997	105.8	6.5
					Bisulfite	98.2	0.991	102.8	4.2
		UQ09	9	HEX	Genomic	91.5	0.998	99.2	8.1
					Bisulfite	92.1	0.996	103.0	5.0
	175	UQ14	14	Texas Red	Genomic	94.2	0.998	96.8	3.5
					Bisulfite	92.6	0.992	112.1	8.5
		UQ11	11	CY5	Genomic	94.1	0.997	100.1	4.2
					Bisulfite	92.8	0.992	100.7	6.1
4-plex	125	UQ02	2	Texas Red	Genomic	99.1	0.997	96.6	8.2
		UQ09	9	CY5	Genomic	101.7	0.996	103.3	4.5
	175	UQ14	14	HEX	Genomic	99.2	0.996	102.2	2.7
		UQ11	11	FAM	Genomic	102.1	0.997	108.5	2.6

### Evaluating DNA fragmentation model with universal PCR quantitation assays

The [long]/[short] ratios of any two target region lengths can be determined by applying the following equation to fragment size distribution data (Eq. 3):

3 $${\text{[long]/[short]}} = \frac{{\mathop \sum \nolimits_{{f = b{ }}}^{n} \frac{{{\text{f }}{-}{\text{ b }} + { }1}}{{\text{f }}}{ }C_{f} }}{{\mathop \sum \nolimits_{f = s}^{n} \frac{{{\text{f }}{-}{\text{ s }} + { }1}}{{\text{f }}}{ }C_{f} }},$$

where b is the length of the longer region and s is the length of the shorter region.

**Figure 3:**
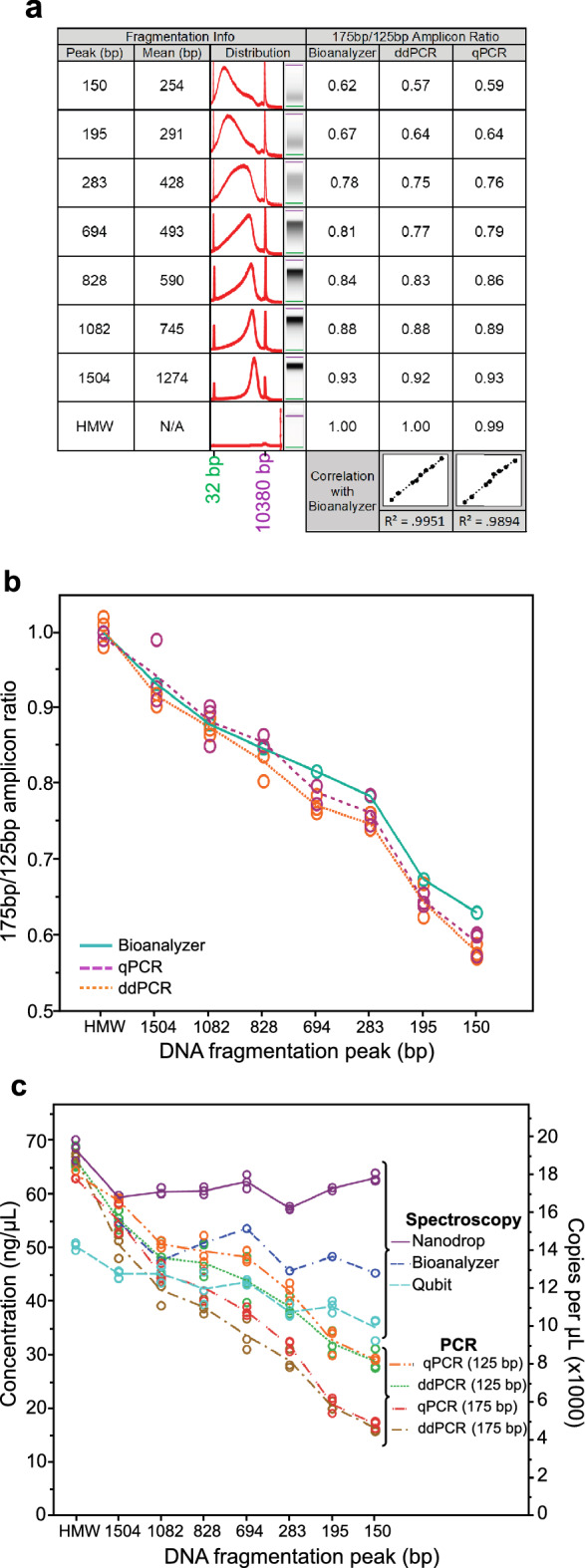
Modelling and quantifying randomly fragmented DNA. (**A**) Table showing DNA samples sonicated to different fragment lengths, their fragment distribution profiles in electropherogram and pseudo-gel form, and comparison between the theoretically (Eq. 3 applied to Bioanalyzer data) and experimentally determined [175 bp]/[125 bp] differential amplicon length ratios. The full unedited pseudo-gel image for these sonicated samples can be found in S6 Fig. (**B**) Line graph plotting the [175 bp]/[125 bp] ratios determined by qPCR, ddPCR, and our mathematical model applied to fragment size distribution data (Bioanalyzer) on differentially fragmented DNA samples. (**C**) Comparison of nucleic acid quantification methods on fragmented DNA. PCR data points are averages of the two universal assays for each amplicon length per well. Fluorometric (Qubit) and absorbance (Nanodrop) spectroscopy measurements were made on each sample on three separate occasions. Spectroscopy concertation measurements are depicted in ng/µl and PCR as copies/µl. Axes are scaled so that 1 copy = 3.5 pg.

To evaluate how well our model of stochastic fragmentation fit with experimental results we compared [175 bp]/[125 bp] ddPCR and qPCR ratios with those derived using Eq. (3) on Agilent 2100 Bioanalyzer fragment size concentration data. This analysis was performed on seven levels of increasing fragmentation induced by the ultrasonication of pooled buffy coat gDNA. The ddPCR and qPCR [175 bp]/[125 bp] ratios of our sonicated samples both showed high goodness-of-fit for ratios derived using Eq. (3), with R-square values of 0.995 and 0.989 for ddPCR and qPCR, respectively (Fig. 3A,B).

### Effects of fragmentation on DNA quantification

Quantification of DNA samples affects all subsequent experimental steps and can lead to costly experimental failures if this step is not performed accurately. Therefore, to further extend our study we next compared the effects of fragmentation on nucleic acid quantification techniques using our sonicated DNA samples, referred to here by their peak (modal) fragment sizes: 150, 195, 283, 694, 828, 1082, and 1504 bp.

One overlooked aspect of DNA fragmentation is that it results in fewer adjacent base pairs for fluorescent DNA dyes to intercalate when dye-based fluorometric methods are used. Thus predictably, and as other studies have noted^1,2^, the mean DNA concentration measured by fluorescence spectroscopy (Qubit 2.0) decreased with increasing fragmentation (*p* < 0.001; one-way ANOVA), with untreated gDNA measuring at 50.40 ng/µl (SD = 0.72), and the most fragmented sample (150 bp) at 35.27 ng/µl (SD = 2.14), which calculate to 14,400 (SD = 206) and 10,100 (SD = 613) genome copies, respectively, assuming 1 genome weighs 3.5 pg based on the following formula:

4 $$\begin{aligned} {\text{Amount}} \left( {{\text{pg}}} \right) & = \frac{{{\text{length }}\left( {{\text{bp}}} \right)*{\text{pg}}/{\text{g}}*{\text{weight}}\;{\text{of}}\;{\text{bp}} \left( {{\text{g}}/{\text{mole}}/{\text{bp}}} \right)*{\text{copies }} \left( {{\text{molecules}}} \right)}}{{{\text{Avogadro's}}\;{\text{number }} \left( {{\text{molecules}}/{\text{mole}}} \right)}} \\ Amount \left( {pg} \right) & = \frac{{3,234,830,000* 10^{12} *650*1}}{{6.022*10^{23} }} \\ \end{aligned}$$

For absorption spectroscopy (Nanodrop 1000), the mean measurement for intact gDNA was 68.40 ng/µl (SD = 1.97), which calculates to 19,600 (SD = 563) genome copies. Although there was no dose-dependent trend towards decreasing concentration with increasing fragmentation, a one-way ANOVA did show a significant difference in concentration (*p* < 0.001), and a Tukey's HSD test found the concentration of intact gDNA to be significantly higher than all seven levels of fragmentation (*p* < 0.001). The highest mean concentration measured for the fragmented gDNA was 63.10 ng/µl (SD = 0.79; 150 bp) and the lowest was 57.43 ng/µl (SD = 0.32; 283 bp), which calculate to 18,100 (SD = 226) and 16,400 (SD = 92) genome copies, respectively.

Both qPCR and ddPCR measured substantial downward trends in concentration with increasing fragmentation (Fig. 3C). This decline in amplifiable copies with increasing fragmentation reflects an increasing number of breakages in the targeted regions, the magnitude of decline being greater for the 175 bp amplicon as longer target regions are more likely to be cleaved. ddPCR on the intact gDNA measured 18,984 (SD = 765) and 19,058 (SD = 608) mean copies for the two 125 bp assays and 18,905 (SD = 308) and 19,306 (SD = 246) for the two 175 bp assays.

The mean absorbance spectroscopy estimate for the number of genome copies in our intact gDNA sample was only 2.8% greater than the combined mean of the four ddPCR assays (M = 19,063, SD = 150). Whereas, the mean number of genome copies estimate for fluorescence spectroscopy was 25% lower, suggesting this method also underestimated intact, not just fragmented, DNA concentration. Our results, therefore, show that absorbance spectroscopy is the most accurate method for quantifying overall nucleic acid concentration, regardless of the degree of fragmentation. However, this technique lacks sensitivity and becomes increasingly inaccurate at the lower end of its analytical range (1–5 ng/µl)^24^. Absorbance spectroscopy is also highly susceptible to reporting falsely high concentrations due to protein contamination and/or phenolic compounds that absorb UV. PCR-based quantification is highly sensitive and most accurately measures the amount of amplifiable DNA at the amplicon length used. Our universal multiplex assay and accompanying online tool Fragment Calculator, which we detail in the following section, extends this ability to estimate the amount of amplifiable DNA of any given region length, while also providing an estimate of overall concentration when working with human genomic or bisulfite-converted DNA.

### Fragment calculator

In addition to describing the fragmentation of the sample, the dual 175 and 125 bp assays, combined with representative DNA samples, can also be leveraged to estimate the concentration of any other sized DNA region. To better enable this we designed the Fragment Calculator online tool to provide a more quantitative and actionable estimate of fragmentation (www.primer-suite.com/fragcalc). This tool uses measured 175 bp and 125 bp concentrations and the [175 bp]/[125 bp] ratio to estimate the average fragment length of a genomic or bisulfite-converted human DNA sample, the total number of genome copies in a measured sample, as well as the number of amplifiable (unbroken) instances of a DNA region of any length. This tool uses the fragment size distribution data of our seven sonicated DNA samples with average fragment lengths of 254, 291, 428, 493, 590, 745, and 1274 bp, a highly fragmented FFPE DNA sample with an average fragment length of 92 bp to represent the lower bounds of random fragmentation, and four gDNA samples with average fragment lengths of 6714, 15,422, 34,625 and 41,496 bp for the upper bounds (S1 File).

**Figure 4:**
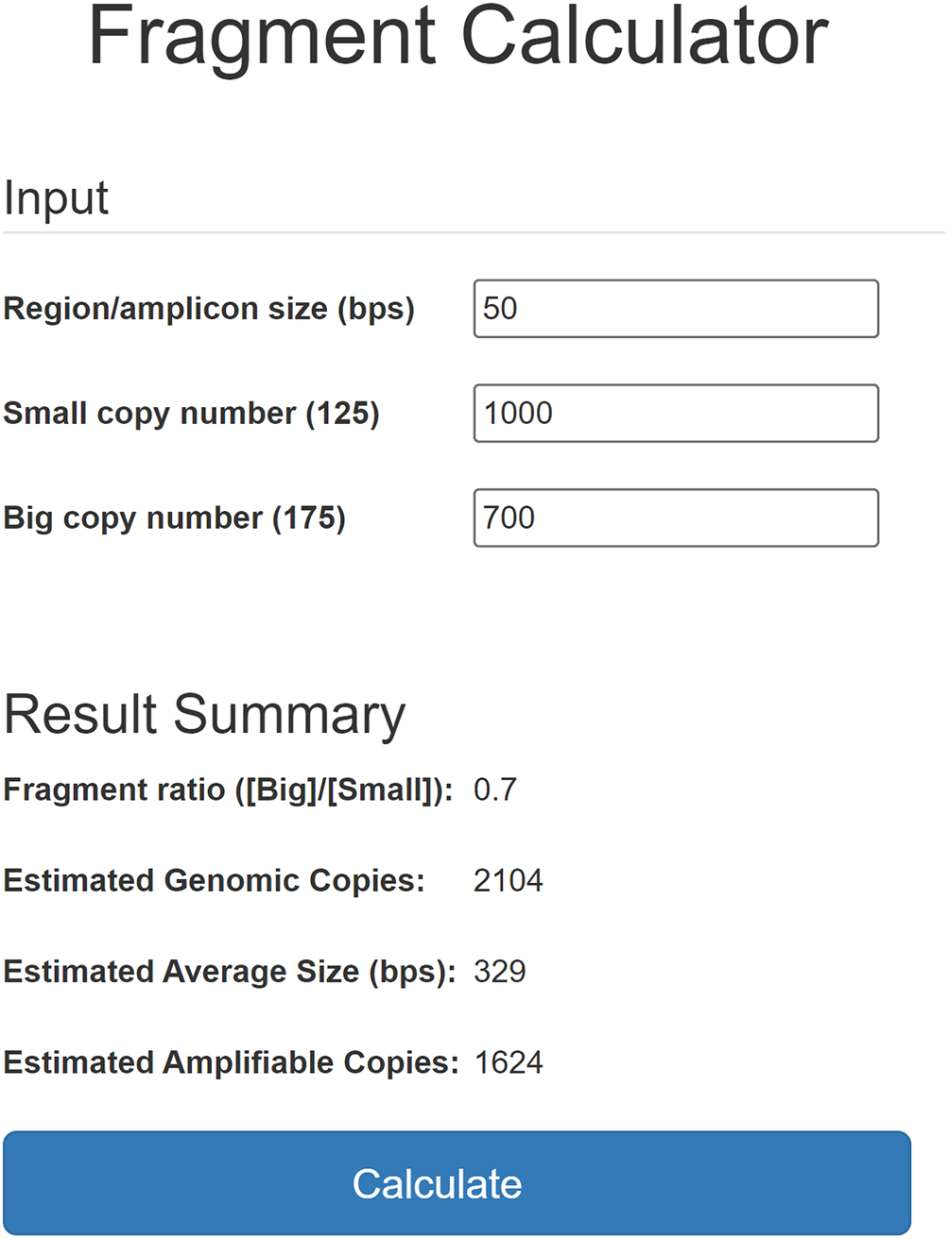
Fragment Calculator online tool with example inputs. The concentrations measured by the two amplicon sizes of our universal quantitative PCR 4-plex assay (125 bp and 175 bp) can be used to estimate the total concentration (i.e., the number of genomic copies), average fragment length of the sample, and the concentration of intact copies of any input region size.

The number of intact copies of an input DNA region length is estimated by taking the two [175 bp]/[125 bp] ratios from our representative fragment size distribution data that an input [175 bp]/[125 bp] ratio falls between (x1, x2), calculating the corresponding [125 bp]/[input size] ratios using Eq. (3) on these size distribution data (y1, y2), determining the slope between these points to estimate the [125 bp]/[input size] ratio corresponding to the input [175 bp]/[125 bp] ratio, and dividing the 125 bp concentration by this ratio. For example, if the concentration measured for a fragmented DNA sample is 1000 copies for the 125 bp amplicon and 700 copies for the 175 bp amplicon, the input [175 bp]/[125 bp] ratio is 0.7, which falls between the [175 bp]/[125 bp] ratios of the 291 bp (0.669) and 428 bp (0.778) reference samples. To estimate the concentration of a 50 bp region, for example, the corresponding [125 bp]/[50 bp] ratios determined using Eq. (3) are 0.585 and 0.707, for the 291 bp and 428 bp reference samples, respectively. The 50 bp concentration is then calculated using the following linear equation:

5 $${\text{y}} = mx + y_{0} ,$$

$$m = \frac{{y_{2} - y_{1} }}{{x_{2} - x_{1} }}$$

$$m = \frac{{0.707 - 0.585{ }}}{0.778 - 0.669}$$

$$[125{\text{bp}}]/[50{\text{bp}}] = 1.119*0.7 - 0.164,$$

$$[50{\text{bp}}] = \frac{{[125{\text{bp}}]}}{{[125{\text{bp}}]/[50{\text{bp}}]}},$$

$$[50{\text{bp}}] = \frac{{1000 \;{\text{copies}}}}{0.619},$$

$$[50{\text{bp}}] = 1615 \;{\text{copies}},$$

where m is the slope and y_0_ is the y-intercept. The number of genome copies is also estimated using this same method by dividing the input 125 bp concentration by the [125 bp]/[1 bp] ratio. Similarly, the average fragment length is estimated using the [175 bp]/[125 bp] ratios from our fragment size distribution data (x1, x2) and their corresponding average fragment lengths (y1, y2) (Fig. 4).

Importantly, Fragment Calculator assumes fragment distributions for the samples being estimated to be similar to those of our representative samples. However, in our experience working with these assays, we have found FFPE samples do not behave like untreated DNA samples. The [175 bp]/[125 bp] ratio for FFPE samples is generally much lower than the ratio calculated from the size distributions of these samples using Eq. (3). This reveals that there is generally less amplifiable DNA in FFPE samples than their size distribution profiles suggest, which we hypothesise is likely due to a combination of single-stranded breaks and incomplete reversal of DNA crosslinking. Our assays are, therefore, a better indicator of the amount of amplifiable FFPE treated DNA than fragment size distribution data from microfluidic capillary electrophoresis instruments like the Agilent 2100 Bioanalyzer.

Further complicating this, however, is evidence that even regions of the same length can have substantially different concentrations of amplifiable FFPE treated DNA. Some of our routine quality control and quantification analyses of FFPE treated samples have revealed vast differences in the number of copies measured by the two 125 bp assays, and these differences are consistent among numerous FFPE samples (S2 File). Despite assays having the same length amplicons, differences in the number of amplifiable copies are likely to occur at high degrees of fragmentation, for instance, due to differences in binding efficiencies among primers when their target regions are truncated. Indeed, we regularly observe statistically significant differences in the number of copies measured by assays of the same size in highly fragmented pooled buffy coat gDNA samples subjected to ultrasonication, some examples of which are forthcoming. However, these differences are relatively small in magnitude and may be due to sequence-specific biases in sonication-induced scission^25,26^. We hypothesise that the much greater differences we observe in FFPE samples may emerge due to differences in the degree to which crosslinking is reversed among regions, as well as potential differences in their susceptibility to DNA breakage. These differences may reflect an underlying nucleosome footprint given that formaldehyde cross-linking is more efficient in nucleosome-bound DNA, as evidenced by the FAIRE-Seq (Formaldehyde-Assisted Isolation of Regulatory Elements) technique^27^.

### Universal multiplex comparison of bisulfite conversion kits

Since PCR-based assays that target both genomic and bisulfite-converted DNA provide more accurate measures of bisulfite conversion recovery than other quantification techniques^28^, we next assessed the performance and utility of our universal multiplex assay to compare the recovery and degree of fragmentation of three commonly used commercial bisulfite conversion kits (MethylEasy Exceed, EZ DNA Methylation-Gold, and EZ DNA Methylation-Lightning) across three starting concentrations (500, 50 and 5 ng) using high molecular weight (HMW) gDNA.

**Figure 5:**
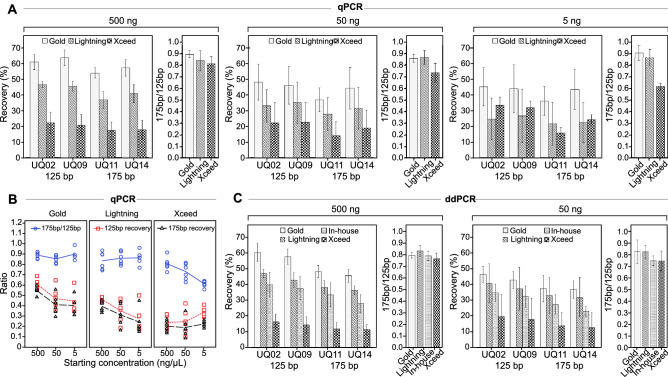
Universal assay comparisons of DNA recovery and fragmentation by bisulfite conversion kits. (**A**) Recovery and fragmentation across different starting concentrations as measured by universal quantitation assays in 4-plex qPCR. (**B**) Plots comparing the recovery and fragmentation trends from qPCR data across decreasing starting concentrations. Recovery data points are averages of the two universal assays for each amplicon length and these values were divided to determine the [175 bp]/[125 bp] ratios. (**C**) Recovery and fragmentation measured by ddPCR 4-plex. Also includes results from in-house bisulfite protocol. (**A**, **C**) Each conversion was conducted in six replicates per concentration for each kit. [175 bp]/[125 bp] fragmentation ratios were calculated by dividing the average copies of the two 175 bp assays by the average of copies the two 125 bp assays. Error bars represent one standard deviation.

A three-way ANOVA on the qPCR results found significant effects of starting concentration (*p* < 0.001), assay (*p* < 0.001), and conversion kit (*p* < 0.001) on recovery (Fig. 5A). Additionally, a significant interaction was found between starting concentration and kit (*p* < 0.001), resulting from an increase in recovery with decreasing concentration in MethylEasy Exceed but a decrease in EZ DNA Methylation-Gold and EZ DNA Methylation-Lightning. Trends were similar for the 125 bp and 175 bp assays, except in MethylEasy Xceed where the proportional increase in mean recovery between 50 and 5 ng was greater in 125 bp assays (22%, SD = 12 vs. 32%, SD = 5) compared to the 175 bp assays (16%, SD = 10 vs. 20%, SD = 5; Fig. 5B). As for fragmentation, a two-way ANOVA found a significant effect of conversion kit on the [175 bp]/[125 bp] ratio (*p* < 0.001), no significant effect of starting concentration (*p* = 0.251), but a significant interaction between kit and concentration (*p* = 0.027) arising from a decrease in the [175 bp]/[125 bp] ratio of MethylEasy Xceed with decreasing starting concentration.

Due to the low starting concentration and recovery of the 5 ng samples, we did not have enough sample left for ddPCR analysis and therefore only ran the 500 ng and 50 ng samples. In addition to the three commercial kits, we also included our in-house bisulfite conversion protocol in these ddPCR comparisons (Fig. 5C). A three-way ANOVA showed similar results to the qPCR analysis, with significant effects of starting concentration (*p* < 0.001), assay (*p* < 0.001), and conversion kit (*p* < 0.001) on recovery, and a significant interaction between kit and concentration (*p* = 0.001). Similar to qPCR, this interaction resulted from declines in the mean recovery of similar proportions between 500 and 50 ng in all kits except MethylEasy Xceed, which showed a mild increase (13%, SD = 4 vs. 16%, SD = 11). A two-way ANOVA found a slight statistically significant difference in the [175 bp]/[125 bp] ratios among conversion kits (*p* = 0.033), which a Tukey's HSD test showed resulted from a significant difference (*p* = 0.048) between DNA Methylation-Lightning (M = 0.83, SD = 0.05) and MethylEasy Xceed (M = 0.75, SD = 0.07). Our in-house method and DNA Methylation-Gold had mean ratios of 0.77 (SD = 0.04) and 0.81 (SD = 0.07), respectively. To estimate the absolute nucleic acid recovery and average fragment size after bisulfite conversion we used our Fragment Calculator tool on combined qPCR and ddPCR results (Table 2).

**Table 2:** Recovery and fragmentation of bisulfite kits using universal quantification 4-plex.

Sample (ng)	Conversion Kit	175 bp % recovery^a^	125 bp % recovery^a^	[Big]/[Small]	Total % Recovery^b^	Average size (bp)^b^
500	EZ DNA Methylation-Gold	52 ± 6	61 ± 5	0.85	90	635
	EZ DNA Methylation-Lightning	38 ± 5	45 ± 3	0.84	69	574
	MethylEasy Xceed	15 ± 6	19 ± 7	0.79	31	467
50	EZ DNA Methylation-Gold	39 ± 9	46 ± 9	0.84	68	617
	EZ DNA Methylation-Lightning	31 ± 11	36 ± 12	0.85	54	636
	MethylEasy Xceed	15 ± 9	21 ± 12	0.72	41	362

^a^Combined average of qPCR and ddPCR of both assays of the same length; ± , standard deviation.

^b^Estimate using Fragment Calculator tool.

### Effects of amplicon size and nucleosome positioning on PCR-based cfDNA quantitation

Snyder et al. (2016)^11^ identified nucleosome protection peaks using deep sequencing of pooled cfDNA samples. Implicit in these analyses is the fact that nucleosome position correlated with the enrichment of fragments at specific locations, which could only occur if nucleosome positions were at least somewhat conserved among people. However, it was unclear the extent to which these peaks might shift between individuals. If little movement occurs and peaks are instead universally conserved, this would have important implications for assay design. Targeting such peaks would maximise an assay’s sensitivity in cfDNA while failing to consider nucleosome protection could severely reduce sensitivity.

Snyder et al. (2016)^11^ calculated a Windowed Protection Score (WPS) for each nucleotide position within the mappable human genome by summing the number of sequenced 120–180 bp cfDNA fragments that wholly overlap a centred 120 bp window and subtracting the number that truncate within this window. Peaks in nucleosome-mediated protection were then called by identifying contiguous regions of elevated WPS. Using the nucleosome protection peaks determined for the pooled healthy sample CH01, we designed two cfDNA assays targeting nucleosome protection peaks that could also be used for bisulfite-converted DNA material: a 95 bp assay targeting chromosome 2 (cfUQ02) with an above-average WPS of 108 and maximum distance of 62 bp from the local maxima, and a 100 bp assay targeting chromosome 11 (cfUQ11) with a below-average WPS of 30 and a maximum distance of 56 bp. The mean WPS of the nearly 13 million peaks identified in the CH01 sample is 63.7 (SD = 41.4). We also designed several staggered PCR assays of varying lengths to flank each of these regions.

**Figure 6:**
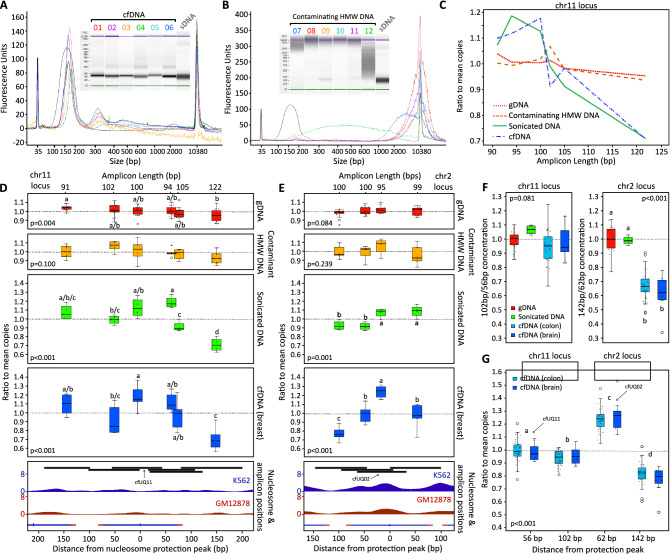
Effects of amplicon length and distance from nucleosome protection peak on intact copies in cfDNA. (**A**–**B**) Electropherograms and pseudo-gel images from Agilent 2100 Bioanalyzer with a High Sensitivity DNA Chip (2100 Expert version B.02.10.SI764). DNA samples are from plasma of breast cancer patients, except sample labelled sDNA which is a pooled buffy coat gDNA sample sonicated and gel-purified to produce a similar fragment size distribution to cfDNA. Samples classified as true cfDNA samples (**A**) were isolated using our in-house phenol–chloroform method (1–4) and QIAamp Circulating Nucleic Acid Kit with EconoSpin All-In-One Mini Spin Columns (Epoch Life Sciences) instead of columns supplied with the kit (5–6). Samples classified as contaminating buffy coat DNA (**B**) were isolated using QIAamp Circulating Nucleic Acid Kit (7–11) and in-house phenol–chloroform method (12). (**C**–**E**) Plots of ratio to mean copies (assay/sample mean) against amplicon length (**C**) and against distance from nucleosome protection peak (**D**) for assays targeting chr11 nucleosome protection peak locus, and against distance from nucleosome protection peak for assays targeting chr2 locus (**E**). Box and whisker plots are centred above corresponding amplicon positions for each assay, along with cell line data of nucleosome signal (K652 and GM12878) from Kundaje et al. (2012)^29^ and nucleosome protection peak position (blue tick) from Snyder et al. (2016)^11^, adjoined by characteristic 146 bp nucleosomal DNA length (blue) and 10 bp linker DNA (red). Sample numbers for each sample type are gDNA = 15, buffy coat DNA = 6, breast cancer cfDNA = 6, and sonicated DNA = 1 (four technical replicates). (**F-G**) Box and whisker plots for chr11 102 bp/56 bp and chr2 142 bp/62 bp differential distance from protection peak copy count ratios (**F**), as well as the ratio to mean copies across the two loci for cfDNA samples (**G**). Sample numbers for each sample type are gDNA = 20, colon cancer cfDNA = 34, brain cancer cfDNA = 10, and sonicated DNA = 1 (four technical replicates). All four amplicons are 100 bp in length. Letters above or below box and whisker plots (**D**–**G**) represent homogenous subsets determined by post hoc Tukey’s HSD analyses (α = 0.05) of one-way ANOVAs (p values on plots). The bottom line of each box represents the 25th percentile, top line the 75th percentile, and thick middle line the median. Whiskers extend up to a maximum of 1.5 times the height of the box. Any values that fall outside this range are classified as outliers (circles). Values that are greater than 3 times the height of the box are classified as extreme outliers (asterisks).

15 cfDNA samples isolated from the blood plasma of breast cancer patients were profiled using dye-based ddPCR to compare the number of amplifiable copies of our universal cfDNA assays along with these staggered assays. We observed that some samples displayed substantial differences in amplifiable copies among assays whereas others did not and that this appeared to coincide with the technique used for cfDNA isolation. We measured the fragmentation profiles of these samples and found 6 displayed characteristic ~ 166 peaks with no sign of HMW contamination, which we thus classified as true cfDNA (Fig. 6A), 6 had little to no cfDNA peak and were reclassified as contaminating HMW DNA (Fig. 6B), and 3 had strong cfDNA peaks but also possible or likely contamination by HMW DNA and were excluded from analysis (S7 Fig). Although high levels of HMW DNA can occur in cfDNA due to non-apoptotic cell death (e.g., necrosis), we suspect the source in these samples was instead the result of poor plasma separation and extraction. Regardless of its source, we only expect to find nucleosome-mediated patterns of fragmentation in the DNA of apoptosed cells, and HMW DNA is likely to obscure these patterns.

To normalise among samples of the same category the concentration measured for each assay was divided by the mean concentration of all assays within each region (chr11 or chr2), giving a ratio to mean copies (assay/sample mean). For ddPCR on HMW gDNA, all assays specific for unique regions should measure the same number of copies within the same sample. Therefore, the ratio of copies measured for a single assay to the mean copies of all assays should be 1:1 for intact gDNA, regardless of proximity to nucleosome peaks. Consistent with this, a one-way ANOVA on samples classified as contaminating HMW DNA found no statistical difference in ratio to mean copies among assays in the chr11 (*p* = 0.100) and chr2 (*p* = 0.239) regions. HMW gDNA samples extracted from the blood of 15 healthy individuals were also used as negative controls and similarly showed little variation in ratio to mean copies among assays. No significant difference was found among assays in the chr2 region (*p* = 0.084). However, a significant difference was detected in the chr11 region (*p* = 0.004), resulting from a minor effect of amplicon length on the number of amplifiable copies (Fig. 6C). A similar trend appears to exist in the contaminating HMW DNA; however, its effects likely did not reach statistical significance due to the smaller sample size (6 vs. 15).

In contrast, the ratio to mean copies for cfDNA decreased with increasing distance from the nucleosome peak, with the highest ratio for each region being our universal cfDNA assays (cfUQ11 and cfUQ2). However, given that cfDNA is highly fragmented, differential amplicons sizes are likely to result in differences in the number of amplifiable copies, therefore confounding the effects of nucleosome protection. To control for this we used ultrasonication and gel purification to produce a blood pooled gDNA sample with a similar level of fragmentation as cfDNA, which we measured in four technical replicates for each assay to compare the effects of random fragmentation on the number of amplifiable copies. In the chr11 region, which had the greatest variance in amplicon size among assays, similar ratios were observed in the sonicated DNA and cfDNA for each assay tested (Fig. 6D). A two-way ANOVA comparing these two sample types found a significant difference among assays (*p* < 0.001) but no statistically significant interaction between sample type and assay, signifying that only the cfDNA level of fragmentation, and not nucleosome protection, was affecting the number of amplifiable copies (*p* = 0.637). These results show that even small differences in amplicon length can have a significant impact on the number of amplifiable copies at such high levels of fragmentation but proximity to the nucleosome protection peak is likely providing little to no differential protection within this region.

Conversely, the assays targeting the chr2 region were far less variable in length and showed little difference in ratio to mean copies in the sonicated DNA, especially when compared to the cfDNA. A one-way ANOVA on the sonicated samples within this region did find significant differences in concentration ratios among assays (*p* = 0.001); however, the magnitudes of these differences were small, they did not track with differences in amplicon length, and they appear to result from a positional effect, perhaps resulting from a sequence-specific bias in fragmentation within this region. Unlike the chr11 assays, the ratio to mean copies for the chr2 assays tracked the distance from the nucleosome peak in cfDNA, rather than the amplicon length. A two-way ANOVA comparing the sonicated and cfDNA samples found a significant difference among assays (*p* < 0.001) as well as a significant interaction between assay and sample (*p* < 0.001), which supports cfDNA having an effect on the number of amplifiable copies in this region beyond that caused by its level of fragmentation on differently sized amplicons (Fig. 6E). Notably, a one-way ANOVA on the cfDNA samples showed no significant difference (*p* = 0.495) in ratio to mean copies (M = 1.00, SD = 0.10 vs. M = 0.97, SD = 0.13) for the two assays with the most similar maximum distances from the nucleosome peak (92 and 99 bp) and only 1 bp difference in length (100 vs. 99 bp.). Whereas, the 50 bp distance (92 vs. 142 bp) separating the two 100 bp amplicons resulted in a significant decrease (M = 1.00, SD = 0.10 vs. M = 0.78, SD = 0.07; *p* < 0.001), and the 95 bp universal cfDNA assay with the smallest maximum distance from the nucleosome peak (62 bp) had a significantly higher ratio (M = 1.25, SD = 0.06) than each of the other three assays (*p* < 0.001; Tukey’s HSD). Despite HMW contamination, the three samples with substantial cfDNA size peaks excluded from this analysis also revealed differences in copies among assays that match a nucleosome-mediated fragmentation pattern in the chr2 region (S3 File).

To further explore and confirm these results we designed probes for one flanking assay per region (in addition to the probes already designed for the cfUQ11 and cfUQ02 universal cfDNA assays), selecting those with the greatest difference between the sonicated and cfDNA samples. Where necessary, the forward or reverse primer for each assay was redesigned to normalise all amplicons to 100 bp while maintaining the same maximum distance from the nucleosome peak. We ran these assays in duplex ddPCR on cfDNA samples extracted from the blood plasma of 34 patients with colorectal cancer and 10 patients with brain cancer, as well as gDNA samples from the blood of 20 healthy donors and four technical replicates of the sonicated gDNA. We then calculated the ratio of copies for the assay furthest to the assay closest to the nucleosome peak (chr11 = [102 bp]/[56 bp] and chr2 = [142 bp]/[62 bp]) and compared the four sample types for each region. For the chr11 region, a one-way ANOVA found no significant difference between the ratios of the colorectal (M = 0.95, SD = 0.11) or brain cancer (M = 0.98, SD = 0.11) cfDNA, gDNA (M = 1.00, SD = 0.06), or sonicated DNA (M = 1.07, SD = 0.03) samples (*p* = 0.081). Although not significant, these differences tended towards a slight nucleosome-mediated protective effect (Fig. 6F).

Conversely, a one-way ANOVA found a significant difference (*p* < 0.001) among sample types for the chr2 region. A post hoc Tukey HSD test showed this difference was due to a drop in the [142 bp]/[62 bp] ratio in cfDNA, with gDNA (M = 1.00, SD = 0.09) and sonicated DNA (M = 1.00, SD = 0.03) being placed in one homogenous subset, and colorectal (M = 0.67, SD = 0.10) and brain cancer (M = 0.62, SD = 0.12) cfDNA placed in another (*p* < 0.001). These results strongly reinforce our previous findings, showing that, unlike the chr11 nucleosome peak, the chr2 peak provides substantial and consistent protection from fragmentation among individuals. Furthermore, comparison across these two regions revealed that the stronger chr2 protection peak resulted not only in greater protection than the weaker chr11 peak but greater degradation in the adjacent valley (Fig. 6G). A two-way ANOVA found significant differences (*p* < 0.001) in the ratio to mean copies between the four assays, and no significant interaction (*p* = 0.189) between the colorectal and brain cancer samples, indicating that the differences between assays were similar for these two cohorts. A Tukey HSD test showed significant differences between all four assays, with the chr2:142 bp (M = 0.81, SD = 0.09), chr11:102 bp (M = 0.95, SD = 0.06), chr11:56 bp (M = 1.00, SD = 0.08), and chr2:62 bp (M = 1.24, SD = 0.10) assays each being placed into separate homogenous subsets (α = 0.025). These results are consistent with cfDNA protection peaks being the result of nucleosome occupancy. As predicted, the protection peak with a low WPS provided weaker but more even protection within its occupied region and the peak with a high WPS provided greater but more narrow protection, thus validating the WPS metric that Snyder et al. (2016)^11^ applied in their analyses.

## Discussion

DNA fragmentation accrues under a variety of experimental and clinically relevant conditions, including sample fixation (e.g., FFPE) and bisulfite conversion, as well as endemically in cfDNA. Accurate quantification of DNA samples underpins all subsequent experimental steps but is a substantial challenge when working with degraded or highly fragmented DNA. Assays can be highly sensitive to DNA concentration and thus inaccurate quantification can result in costly experimental failures and sample expenditure. Even without experimental failure, overestimating the amount of genetic material required for an assay can result in needless wastage of samples. This is particularly problematic in the case of samples that are precious, limited and/or costly.

Nucleic acid quantification techniques include fluorescence spectroscopy using DNA-binding dyes, ultraviolet–visible absorption spectroscopy, and PCR-based quantification. Fluorescence spectroscopy (e.g., Qubit) is a highly sensitive quantification technique; however, its reliance on calibrations standards means that any inaccuracy in the reported concentration of these standards is propagated into each measurement. This shortcoming is exemplified when measuring fragmented DNA, where the accuracy of fluorescence spectroscopy is diminished by increasing levels of fragmentation, as demonstrated in our results and those of previous studies^1,2^. To avoid this decline multiple calibration standards would need to be used, each matched to the same level of fragmentation as the samples being measured. However, this solution is impractical as it requires the level of fragmentation of a sample to be measured prior to its concentration, as well as the ability to reliably reproduce this level of fragmentation in the calibration standard. As for absorption spectroscopy (e.g., Nanodrop), this technique is both accurate and largely unaffected by DNA fragmentation but lacks sensitivity and requires DNA samples of high purity. Whereas, PCR-based quantification is accurate, highly sensitive and generally less affected by impurities, but measures the amount of amplifiable DNA of a given length, rather than the absolute nucleic acid concentration.

In addition to measuring the concentration of fragmented DNA samples, it is also important to measure the degree of fragmentation, as the amount of useable DNA within a sample will depend on the fragment length required by an assay. For example, a PCR reaction will fail if the length of fragments within the sample are all shorter than the length of the designed amplicon. Unlike conventional agarose gel electrophoresis, microfluidic capillary electrophoresis (e.g., Agilent 2100 Bioanalyzer) provides a highly quantitative analysis of the level of DNA fragmentation and only requires nanograms rather than micrograms of a sample, making it the gold standard for assessing the integrity of nucleic acid samples. However, microfluidics sizing platforms are limited in their capacity for nucleic acid quantification, as they operate within a narrow range of fragment lengths and concentrations. That is, the concentration of a sample must first be measured by an alternative method to determine the input amount, and the fragment size profile of a DNA sample must fall between an upper and lower size marker that encompasses the range of ladder fragment sizes.

In this study, we aimed to produce PCR-based multiplex assays for the accurate quantification of both concentration and the degree of fragmentation of clinical DNA samples. To do so, we investigated the phenomena that inhibit accurate quantification in quantitative PCR and then addressed these in our assay design. We started by addressing the key limitation of PCR-based assays when quantifying randomly fragmented DNA: their measurement of the number of amplifiable copies at a specific target length. The limitation being these techniques do not give a measure of the overall concentration (i.e., the number of genomic copies pre-fragmentation) or the number of amplifiable copies at different lengths. We addressed this limitation by mathematically modelling random fragmentation, designing the amplicons in our multiplex at two different lengths to provide a ratiometric measure of fragmentation, and then demonstrating that our experimentally determined ratios strongly agreed with those derived mathematically. This allows us to use reference samples to estimate the number of amplifiable copies at any given length, as well as the number of genome copies within a fragmented DNA sample.

Another issue with PCR-based quantification is that target regions can undergo copy number variation. In a single target assay, such variation can result in copy number measures vastly discordant with the actual number of genome copies within a sample. To address this, we included two amplicons for each of the two lengths and targeted a separate chromosome for each, thus allowing for cross-checking to confirm that copy number aberrations have not occurred in the target regions. In multiplex, these copy number controls and fragmentation analyses are added while not requiring any additional sample compared to a single target assay. To further control for copy number variation, amplicons were designed to target regions of the genome that rarely undergo copy number aberrations in cancer samples, based on TCGA data from 10,610 patients.

Beyond simple fragmentation, bisulfite conversion causes sample loss and fundamental changes to the DNA sequence, rendering genomic- and bisulfite- based quantification assays incomparable without the ability to normalise bisulfite-converted DNA concentrations to that of their genomic counterpart. In fluorescence spectroscopy, this is hindered by an inability to distinguish the degree to which a drop in concentration post bisulfite conversion is due to fragmentation and how much is due to sample loss. For spectroscopy in general, it is unclear which fluorometric assay or absorbance setting (double-stranded DNA, single-stranded DNA or RNA) best represents bisulfite-converted DNA, which contains the RNA base uracil and a mixture of both double and single strands. We addressed this issue by targeting the primer and probes of our assays to cytosine-free regions, allowing the amplification of both bisulfite-converted and genomic human DNA. We then demonstrated the utility of these assays by conducting performance comparison experiments on three commercial bisulfite conversion kits. Comparisons of commercial bisulfite treatment kits performed on HMW, FFPE and cfDNA samples in previous studies have consistently shown superior performance in recovery, conversion efficiency and relative integrity in EZ DNA Methylation-Gold kits, similar performance in integrity and conversion efficiency but lower recovery in EZ DNA Methylation-Lightning, and poor recovery and conversion efficiency with qualitatively similar levels of fragmentation in MethylEasy Xceed compared to these other two kits^28,30–32^. Our findings corroborated the recovery results of these previous studies, supporting the ability of our universal multiplex assay to accurately measure the concentration of both genomic and bisulfite-converted DNA samples. In addition, our universal multiplex assay provides an alternative to electrophoresis, with quantification performed in conjunction with, rather than a precursor to, fragmentation assessment. Unlike the commonly used qualitative assessment of DNA fragmentation using standard gel electrophoresis, our ratiometric measure allows for a quantitative assessment and statistical comparison between methods. This high sensitivity all-in-one approach makes our universal multiplex assay a cost-effective quantifier of DNA concentration and integrity that requires minimal sample expenditure.

Finally, unlike the fragmentation caused by FFPE, ultrasonication or bisulfite treatment, cfDNA fragmentation is non-stochastic and mediated by protection from endonuclease digestion afforded by histone binding. We, therefore, designed two additional universal quantification assays specifically for cfDNA by targeting nucleosome protection peaks in unique, copy number invariant, cytosine-free regions. To validate these assays and assess the effects of amplicon length and distance from the nucleosome peak, we designed 8 additional flanking assays tiled across these two nucleosome-associated target regions. Overall, our results indicate that the further an assay is from a nucleosome protection peak the lower the number of intact amplifiable copies will exist in cfDNA for its target region and that this is mediated by the strength of the peak (i.e., more conserved nucleosome positions provide greater protection). In the region with the stronger of the two protection peaks (WPS ~ 1 standard deviation greater than average), we found the number of amplifiable copies measured by two overlapping 100 bp amplicons offset by 80 bp differed significantly, with the assay centred on the nucleosome protection peak averaging 1.5 times, and measuring up to 3 times, the number of copies as the flanking assay. It is likely these differences would be even greater near nucleosome peaks with stronger positional conservation. Such differences reveal inherent problems in quantifying cfDNA and its use in various assays, such as copy number variation detection, that must be carefully considered during assay design.

Our two universal cfDNA assays can be used to give a general assessment of concentration for both genomic and bisulfite-converted cfDNA, as they account for both relatively strong and relatively weak nucleosome protection. The stronger assay can also be combined with longer amplicons extending beyond the two most substantial cfDNA size peaks (> ~ 400 bp) to assess cfDNA purity and the degree of contamination by HMW cellular DNA^33^. However, because of the wide variation in base coverage caused by nucleosome-mediated fragmentation, the number of amplifiable copies in specific regions must be assessed independently. Our results also further validate the feasibility of “fragmentomics” as a novel field in biomarker research—an idea first proposed by Ivanov et al. (2015)^34^ that entails approximating expression levels of clinically relevant genes and other regulatory changes using nucleosome positioning, and recently demonstrated using whole-genome cfDNA sequencing by Snyder et al. (2016)^11^ and Ulz et al. (2016)^12^. Our results demonstrate that PCR-based assays could be used in detecting changes in nucleosome positioning in sites of interest. For example, two or more staggered amplicons could be employed to detect nucleosome depletion as a proxy for transcription initiation in an oncogene promoter region.

## Methods

### cfDNA extraction

Brain tumour patient cfDNA samples were isolated using NucleoSnap DNA Plasma Kit (Scientifix) using the manufacturer’s recommended protocol. Colorectal cancer patient cfDNA samples were isolated using the following in-house protocol based on the method described by Hufnagl et al.^35^. Plasma digest was conducted in a DNA lo-bind 5 ml tube with 200 µl 1X Low TE, 900 µl plasma, 110 µl buffer (250 mM EDTA, 750 mM NaCl, 10 mM Tris), 110 µl 10% SDS, and 22 µl Proteinase K (20 mg/ml, NEB). Incubated for 2 h a 56 °C in a water bath, mixing by hand every 30 min. Added 1 volume of 1342 µl of pH 8.0 phenol/chloroform/isoamyl alcohol, added a big drop of silicone into tubes, vortexed 3 s, revert mixed a few times, incubated 5 min at room temperature, centrifuged at 13000 g for 15 min. Removed upper phase containing DNA and transferred into a new Lo-bind 5 mL tube. Added 2 µl Glycoblue, 630 µl of 7.5 M ammonia acetate and 2 volumes 2520 µl cold 100% ethanol, mixing well by inverting the tubes. Tubes were then incubated overnight at -20 °C then spun for 30 min at 12,000 rpm at room temperature to pellet DNA. Pellet was washed twice with 70% ethanol then air-dried and resuspended with 40 µl of 1X Low TE. As mentioned in our results, a portion of cfDNA samples from breast cancer patients was also isolated with this method. The remaining breast cancer cfDNA samples were extracted using QIAamp Circulating Nucleic Acid Kit (Qiagen) using the manufacturer’s recommended protocol, except for those where EconoSpin All-In-One Mini Spin Columns (Epoch Life Sciences) were used rather than the columns supplied in the kit.

### Determining [175 bp]/[125 bp] fragment distribution ratios

Concentrations for each fragment length were derived from Agilent 2100 Bioanalyzer electropherograms. Fragment concentrations were extracted for each sample by setting windows in the Region Table of the Agilent 2100 Expert Software. As the software cannot handle more than 85 regions before crashing, we automated a procedure of extracting the concentrations from each fragment size 85 at a time using Pulover's Macro Creator version 5.0.5. We set the window sizes to 0.6-s increments to match the data points in the 2100 Expert software. These concentrations and their corresponding average fragment lengths were then placed into Eq. (3) detailed in our results to determine the [175 bp]/[125 bp] ratio of each sample.

### PCR assay design

Copy number aberration (CNA) regions were parsed from the CosmicCompleteCNA.tsv version 78 downloaded from http://cancer.sanger.ac.uk19. Regions were filtered using a Python script to exclude duplicates from the same sample, regions from cell line samples (determined by sample names from the Cosmic Cell Lines Project), and those missing total copy number and minor allele counts.

Next, regions were converted into BED format and uploaded to the Galaxy web platform^36^. We used the public server at usegalaxy.org to produce a bedGraph using the Genome Coverage tool from the BEDtools package^37^. This bedGraph was composed of 170,410 regions of varying sizes, each derived by calculating the number of CNA regions overlapping each nucleotide within the genome and merging all adjacent nucleotides with the same number into a single region with a corresponding sample number. This bedGraph was used to make our Circos plot histogram^38^.

We set a threshold at the bottom 10th percentile of regions, excluding those where greater than 34 of the 10,610 samples had significant copy number variation. We then removed regions with greater than 10 kb between Affymetrix Genome-Wide Human SNP Array 6.0 copy number probes (used for TCGA CNA detection) using the Galaxy web platform intersect function to filter out regions with potential artefactually low CNAs. For the cfDNA assays, we produced a BED file with the coordinates of 65 bp either side of the local maxima for each nucleosome protection peak and intersected these with the remaining copy number invariant regions.

Using the getFasta function from BEDtools we extracted the sequences for the copy number invariant regions. We then ran a modified version of our PrimerSuite^39^ primer design algorithm on these sequences to produce all potential 125 bp and 175 bp amplicons for the random fragmentation assays, and all 90–100 bp amplicons for the cfDNA assays, with ~ 63 °C melting temperature (Tm) primers and ~ 67 °C Tm probes devoid of cysteines on the template strand. Each primer pair was then screened using Bowtie 2^40^ version 2.3.0-legacy in paired-end mode for those forming unique single-mapped amplicons in a 1 kb window for both genomic and bisulfite-converted DNA (both GtoA and CtoT converted genomes). The remaining random fragmentation assays were then run through a multiplex design Python script that incorporated our PrimerDimer^41^ algorithm to create a multiplex that included two 125 bp and two 175 bp assays, with each targeting a separate chromosome and minimised potential for primer dimer formation.

### DNA quantification

DNA was fragmented to different lengths using a Covaris S2 Focus Ultrasonicator.

#### Fluorescence spectroscopy

Samples were quantified using a Qubit 2.0 Fluorometer with a dsDNA High Sensitivity Assay Kit following the manufacturer’s protocol. 1 µl of each sample was used per measurement and repeat measurements were made on three separate occasions.

#### Absorbance spectroscopy

Samples were quantified using a Nanodrop ND-1000 Spectrophotometer set to dsDNA sample type. 2 µl of each sample was used per measurement, with three repeat measurements made per sample.

#### Microfluidic capillary electrophoresis

Fragment distribution analysis and quantification were conducted on each sample using an Agilent 2100 Bioanalyzer with a High Sensitivity DNA Chip following the manufacturer’s protocol. All samples were diluted 1/25 to ~ 2 ng/µl.

#### Droplet digital PCR using universal 4-plex

Samples were quantified using a QX100 ddPCR System (Bio-Rad). Reactions were made to a 22 µL total volume in triplicate per sample and consisted of 1 µL of DNA, 7.36 µL H_2_O, 11 µL 2 × Supermix for Probes, 0.44 µL 10 u/ µL HindIII, 2.2 µL 10 × primer/probe 4-plex mix (2.5 µM per primer for 125 bp amplicons, 5 µM per primer for 175 bp amplicons, 1 probe at 1.25 µM and 1 at 0.75 µM per fluorophore [FAM and HEX]), 0.08 µL 5U/µL Hot Start Taq-Polymerase. Droplets were generated using a QX200 AutoDG Droplet Digital PCR System. Cycling conditions were 10 min at 95 °C, followed by 45 cycles of 30 s at 95 °C and 2 min at 60 °C, and ending with a 10 min 98 °C enzyme deactivation step. Concentrations for each assay were determined in QuantaSoft Analysis Pro 1.0.596 by setting wells to Amplitude Multiplex mode and using the 2D Amplitude window. Concentrations for each sample were averaged for the assays with the same sized amplicons.

#### Quantitative real-time PCR using universal 4-plex

Samples were quantified using a CFX384 Touch Real-Time Detection System (Bio-Rad). Reactions were carried out at 15 µL total volume consisting of 5 µL of DNA, 2.65 µL H_2_O, 3 µL 5 × Colorless GoTaq Flexi Buffer (Promega), 2.7 µL 25 mM MgCl_2_, 0.08 µL 10 mM dNTP mix, 1.5 µL 10X primer/probe 4-plex mix (2.5 µM per primer for 125 bp amplicons, 5 µM per primer for 175 bp amplicons, 1.25 µM per probe [FAM, HEX, Texas Red, and Cy5]), 0.08 µL 5U/µL Hot Start Taq-Polymerase. Cycling conditions were 10 min at 95 °C, followed by 40 cycles of 30 s at 95 °C and 2 min at 60 °C. Concentrations for each of the four assays were averaged from 2–4 technical replicates. Three separate qPCR runs were conducted. Standard curves were set up using eight-part 1:2 dilution series from a starting 18,000 copies/µL pooled gDNA sample or a 2000 copies/µL bisulfite conversion of this sample. Initial concentrations of these samples were determined by ddPCR.

### Creating a gDNA sample with cfDNA sized fragmentation

50 µL of blood pooled gDNA (50 ng/µL) was sonicated using a Covaris S2 Focus Ultrasonicator with the manufacturer’s recommended settings for a 150 bp peak. Sonicated DNA was then run in a 2% E-Gel EX agarose (Invitrogen) on an E-gel Power Snap for 15 min. Gel was then cut around sample between ~ 50–170 bp using a UV lamp. The fraction was then purified using QIAquick Gel Extraction Kit (Qiagen) according to the manufacturer’s protocol. The size distribution profile was measured using a 2100 Bioanalyzer (Agilent Technologies) to confirm similar fragmentation to cfDNA samples.

### Bisulfite DNA conversions

Bisulfite conversions of template DNA were conducted with commercial kits using the manufacturer’s recommended protocols. All reactions were eluted in 20 µL of each kit’s respective buffer. Our in-house method was performed using manual protocols reported previously^42^. Conversion took place at 80 °C for 45 min, followed by resuspension in low TE (10 mM Tris–CL, pH 8.0, 0.1 mM EDTA). For each conversion, DNA was first quantified with the Qubit dsDNA BR Assay Kit and between 500 and 5 ng (20 µL of 25 ng/µL, 2.5 ng/ µL or 0.25 ng/µL) of material was bisulfite converted at a time.

Bisulfite recovery was determined using the qPCR (using a gDNA standard curve) and ddPCR protocols detailed above. Concentration measurements by qPCR were corrected by the factor 2 for bisulfite converted DNA as only the sense strand can be amplified by PCR in bisulfite-converted DNA, thus causing a shift of 1 cycle threshold compared to gDNA. Proportion recovered was determined by dividing each bisulfite converted DNA concentration by the concentration of the input gDNA sample at the respective dilution factor, which was measured along with the bisulfite converted samples.

### Statistical analyses

Graphing and statistical analyses were conducted in IBM SPSS Statistics 24. Circos plot was created using Circos software on our Linux server^38^.

### Ethics approval and sources of biological material

The plasma samples from breast cancer patients used in this study were recruited for a National Breast Cancer Foundation (NBCF) adjuvant clinical trial and plasma samples from healthy donors were received from the Red Cross Blood Service, Australia. The use of these human plasma samples was approved by the Bellberry Human Research Ethics Committee (application number 2015–12-817-A-6). The plasma samples from colorectal cancer patients were recruited for a study by the John Hunter Hospital and the use of these samples was approved by the Hunter New England Human Research Ethics Committee (reference number 11/04/20/4.03). Finally, the plasma samples from brain cancer patients were from The Wesley-St Andrew’s Research Institute (WSRI) Tissue Bank, which has ethics approval from the UnitingCare Health Human Research Ethics Committee (HREC). Informed consent for genomic analyses was obtained from all patients and the research detailed in this article was conducted in accordance with the Declaration of Helsinki Ethical Principles for medical research involving human subjects.

## Supplementary Information

Supplementary Information 1.

Supplementary Information 2.

Supplementary Information 3.

Supplementary Information 4.

## Data Availability

All data generated or analysed during this study are included in this published article [and its supplementary information files]. The Fragment Calculator tool is available at www.primer-suite.com/fragcalc/ as a web application.
