# Supplementary material for: Modelling clinical DNA fragmentation in the development of universal PCR-based assays for bisulfite-converted, formalin-fixed and cell-free DNA sample analysis
Source: Sci Rep. 2022 Sep 26;12:16051. doi: 10.1038/s41598-022-18196-7 (PMC9512909; doi:10.1038/s41598-022-18196-7)
Supplement: Supplementary file 4 — Supplementary Information 4. [file 41598_2022_18196_MOESM4_ESM.pdf]

S1 Table.

Cancer samples from COSMIC/ TCGA used for copy number aberration analysis

**Primary site**	**Primary histology**	**Number of Samples**
Adrenal gland	Carcinoma	89
	Pheochromocytoma	170
Biliary tract	Carcinoma	36
Breast	Carcinoma	1063
Central Nervous System	Glioma	1011
Cervix	Carcinoma	298
Endometrium	Carcinoma	554
Eye	Malignant melanoma	76
Haematopoietic tissue	Neoplasm	175
Lymphoid tissue	Neoplasm	46
Kidney	Carcinoma	835
Large intestine	Carcinoma	600
	Not specified/other	8
Liver	Carcinoma	368
Lung	Carcinoma	994
Oesophagus	Carcinoma	183
Ovary	Carcinoma	565
Pancreas	Carcinoma	176
Pleura	Mesothelioma	87
Prostate	Carcinoma	474
Skin	Malignant melanoma	464
Soft tissue	Sarcoma	249
Stomach	Carcinoma	466
Testis	Germ Cell Tumour	149
Thymus	Thymoma	119
Thyroid	Carcinoma	441
Upper aerodigestive tract	Carcinoma	517
Urinary tract	Carcinoma	397
	Total	10610

S2 Table. Universal quantification assays

Name	Oligo	Sequence
UQ02	Forward	AAGGTGTAAAGTGTAGGTGAAGTTATGAAGTA
	Reverse	TACTATCTTTATCTACATTTCATTACCTCCATCTACA
	Probe	CCTCTATCTATACCCCACATCTCCATTT
UQ09	Forward	CATCCCATCCATTCTCCCTCC
	Reverse	GAAAGTGAGTGGATGAATGGGTAGG
	Probe	TCACTAACCTTTCTTCTCCACCCATCATC
UQ11	Forward	TTCTCCATTCTTCATATTCCATTAACCC
	Reverse	GAGATTATTGAGAATGAGAAAATGTGGTATG
	Probe	CCCATTCTCCATTCTCCATACACCATTCT
UQ14	Forward	AGAATGATGGGAATGTTGGGTAGTAG
	Reverse	CAATATTTCCATCCTCATTTCTCTCCTTAA
	Probe	CCTTCACCATCCAACATTCCCATCCTC
cfUQ02	Forward	ATAGGAAATGGGGATTGGTGGTT
	Reverse	CTCCCCTATCCACACATTAAACC
	Probe	TCTAAAATTCCCCCCCCATCACCACA
cfUQ11	Forward	CTTTCTCCATACTCCACTCTCCATATA
	Reverse	ATGGATGATGAAGAATGGAGAATAGA
	Probe	CCATTTTTCAAACACCATTCTCCTTTCTCT

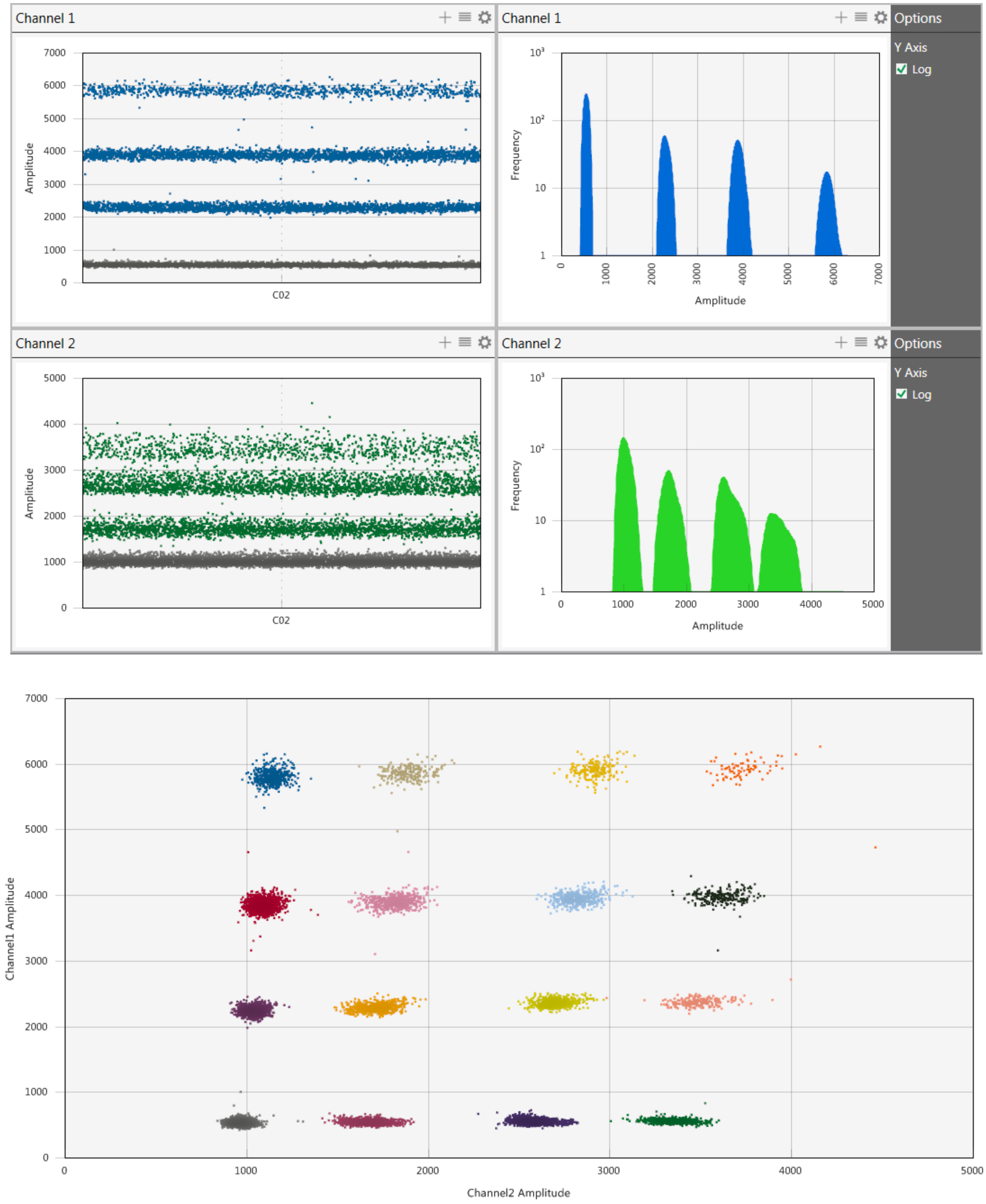

S1 Fig. Example singe channel ddPCR amplitude plots and corresponding two-dimensional amplitude plot from universal 4-plex assay run on pooled buffy coat gDNA sample in a 22 µl reaction. Channel1 plots amplitude from FAM probe florescence and Channel2 from HEX probe florescence. Results obtained from QuantaSoft™ Analysis Pro version 1.0.596.

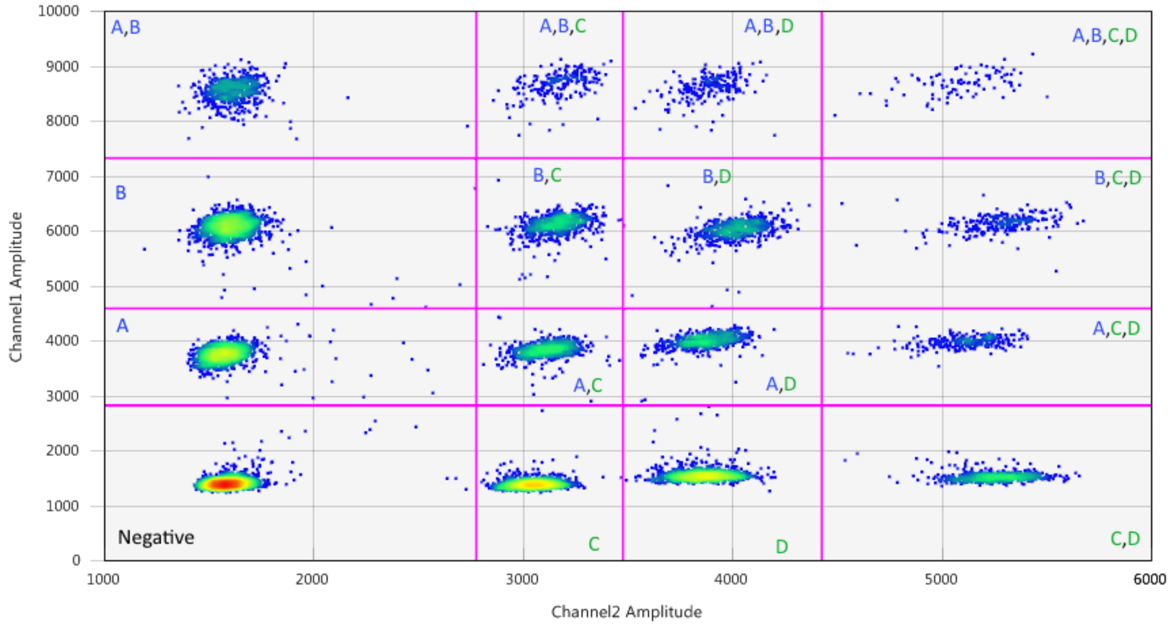

	Well	Target	DyeName(s)	Conc(copies/µL)	PoissonConfMax	PoissonConfMin	Accepted Droplets	Positives	Negatives
A	A01	125bp - chr2	FAM Lo	400	413	388	13250	3823	9427
B	A01	125bp - chr9	FAM Hi	401	414	389	13250	3829	9421
C	A01	175bp - chr11	HEX Lo	419	432	406	13250	3970	9280
D	A01	175bp - chr14	HEX Hi	384	397	372	13250	3693	9557

S2 Fig. Two-dimensional ddPCR amplitude plot with heat map and accompanying results table for universal 4-plex assay run on 25 ng of pooled buffy coat gDNA sample in a 22 µl reaction. Channel1 plots amplitude from FAM probe florescence and Channel2 from HEX probe florescence. Each section is labelled with letters corresponding to the assays represented in the droplets of that section. Results obtained from QuantaSoft™ Analysis Pro version 1.0.596.

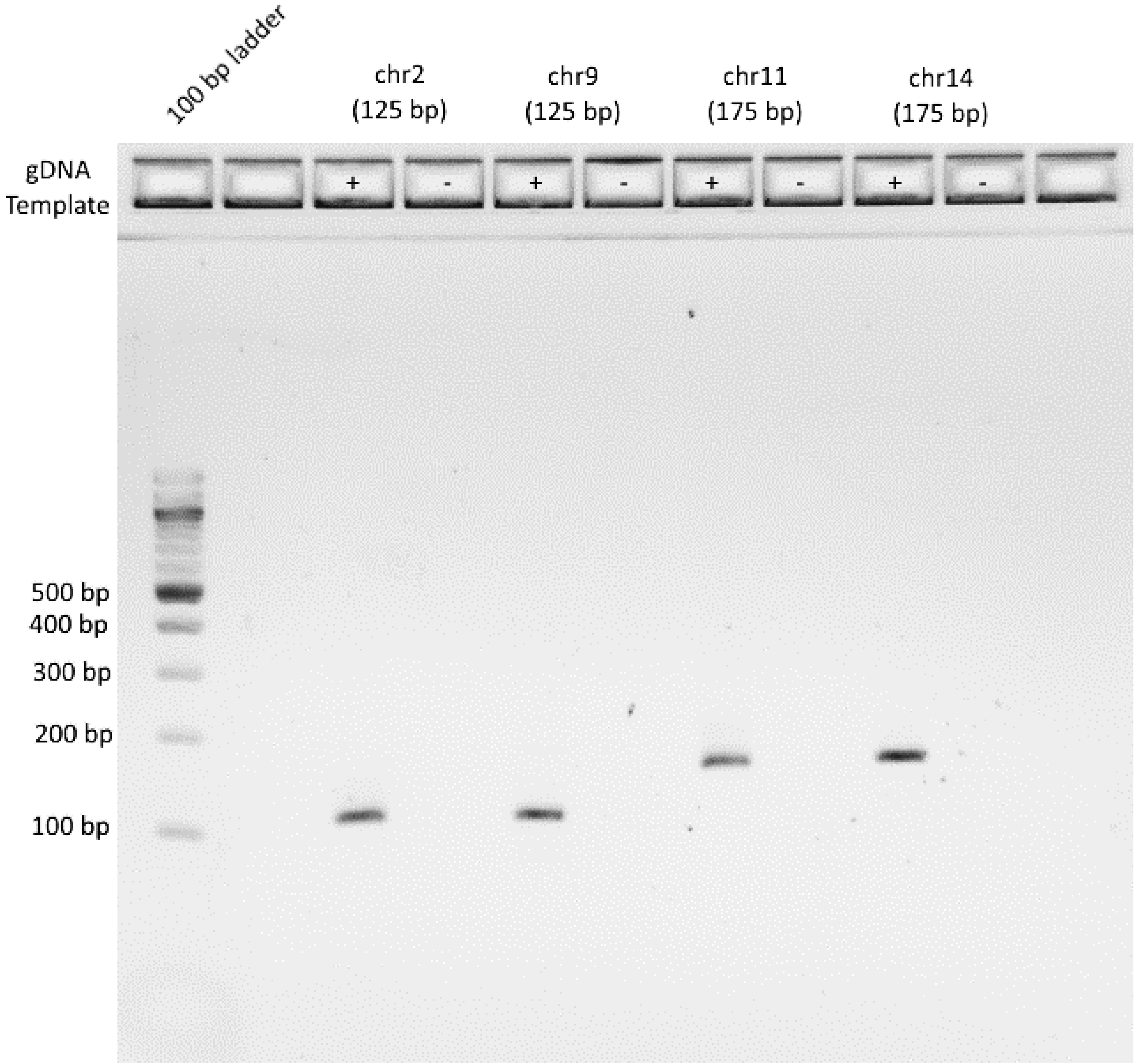

S3 Fig. Gel electrophoresis image of assays from universal 4-plex run individually on pooled gDNA extracted from the buffy coat of 40 healthy donors. Invitrogen 2% Agarose E-Gel. Cycling conditions were 10 minutes at 95°C, followed by 30 cycles of 30 sec at 95°C and 2 min at 60°C.

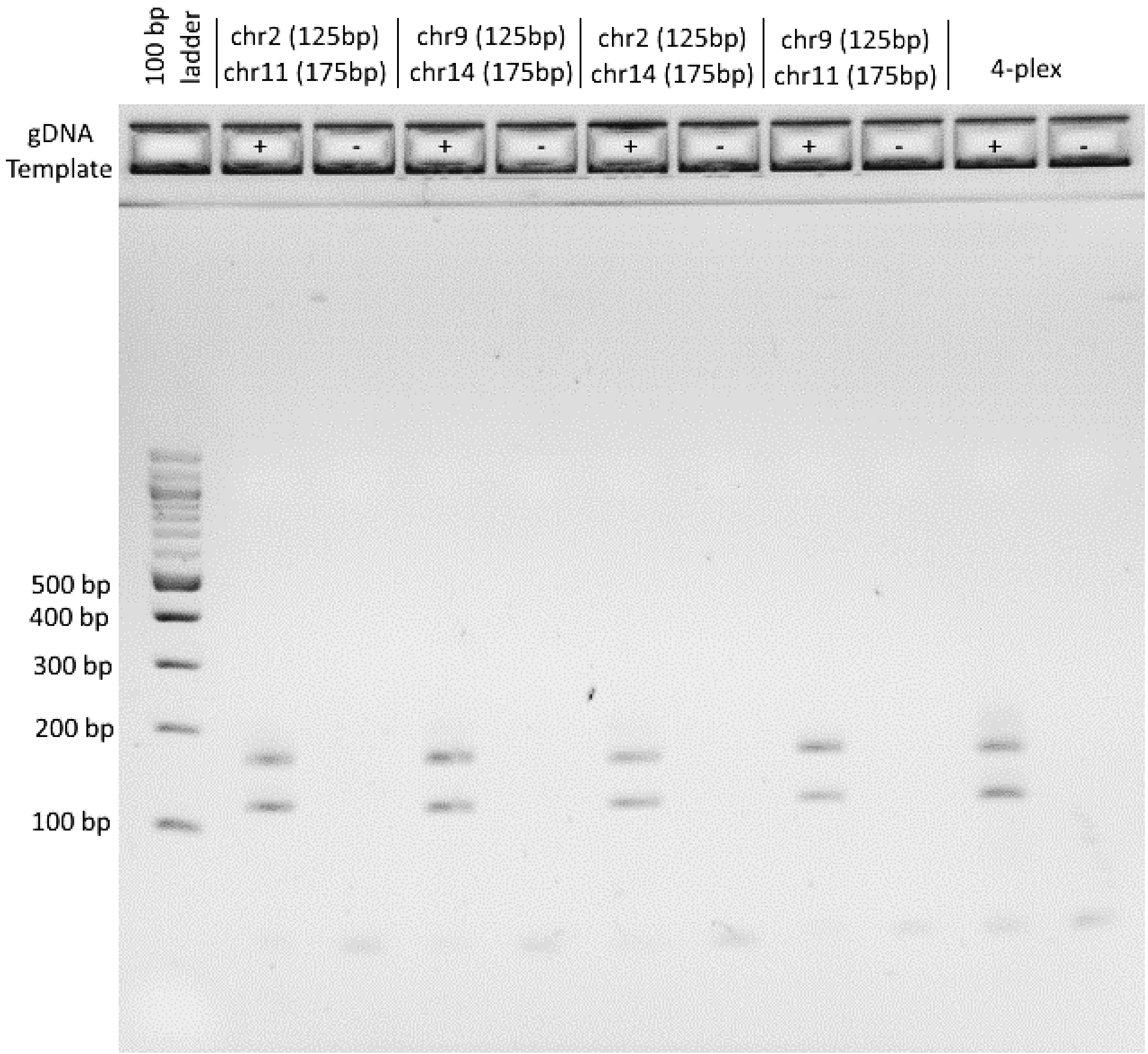

S4 Fig. Gel electrophoresis image of universal assays run in duplex and 4-plex on pooled gDNA extracted from the buffy coat of 40 healthy donors. Invitrogen 2% Agarose E-Gel. Cycling conditions were 10 minutes at 95°C, followed by 30 cycles of 30 sec at 95°C and 2 min at 60°C.

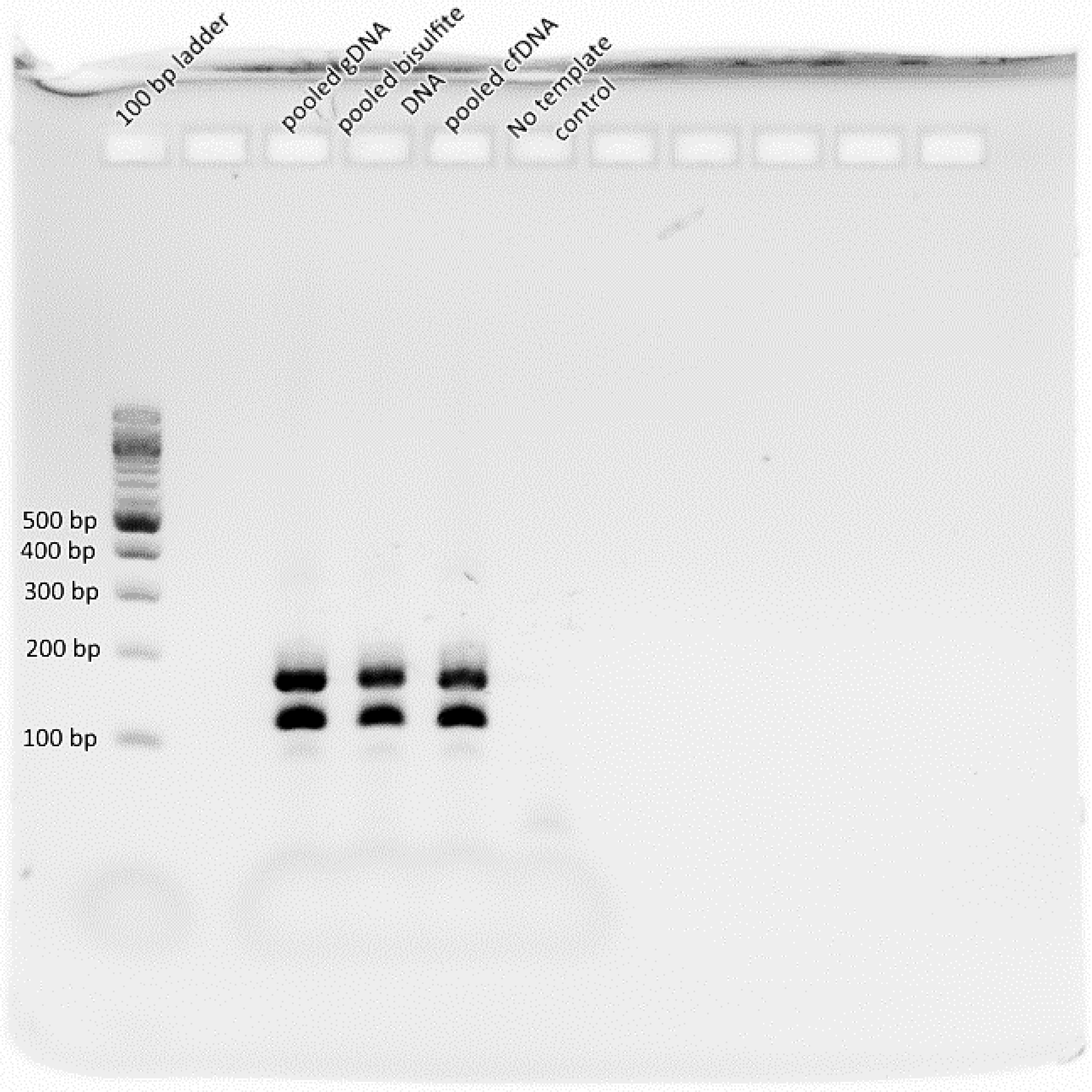

S5 Fig. Gel electrophoresis image of universal 4-plex run on multiple DNA sample types. Multiplex includes two 125 bp and two 175 bp amplicons. Invitrogen 2% Agarose E-Gel. Cycling conditions were 10 minutes at 95°C, followed by 35 cycles of 30 sec at 95°C and 2 min at 60°C.

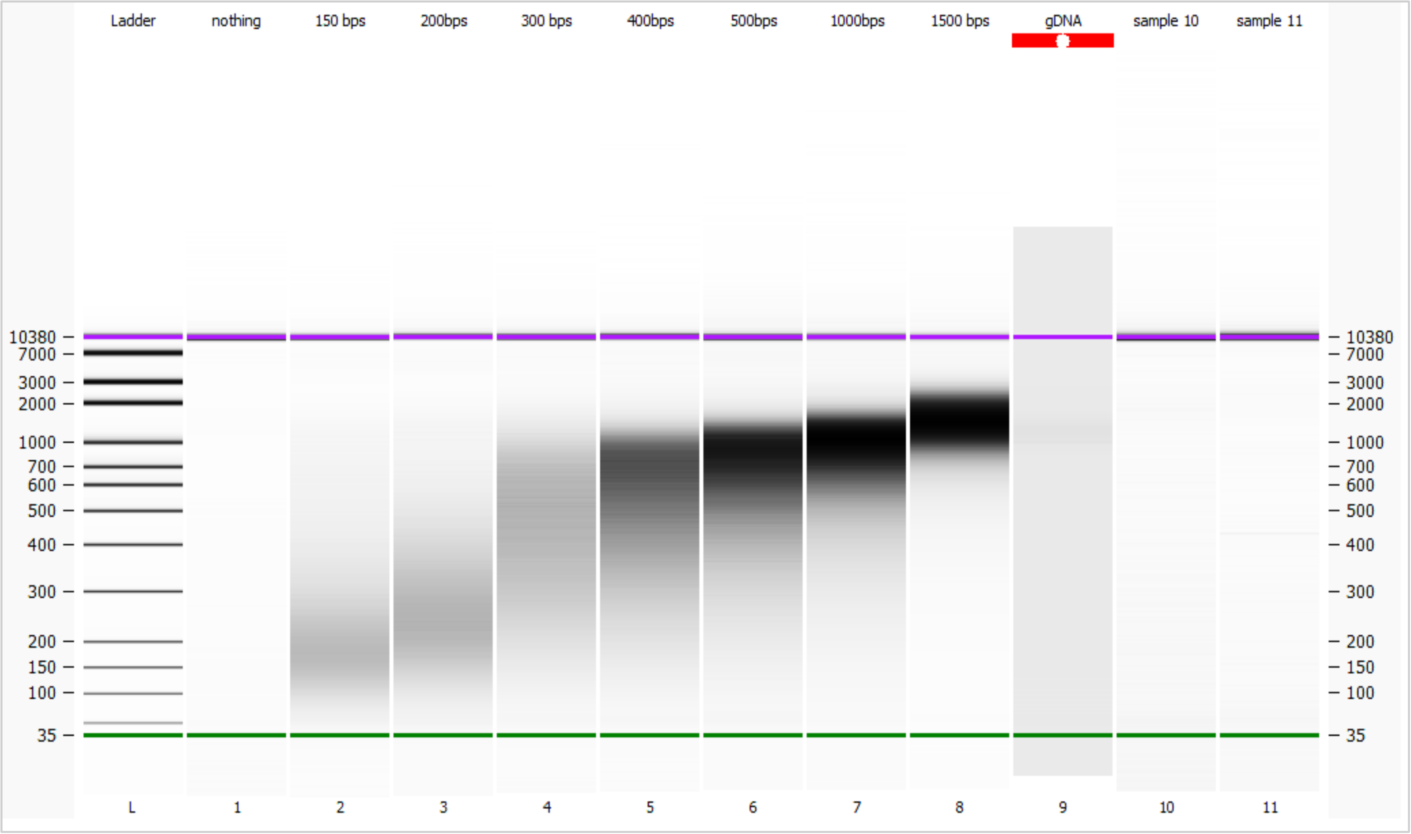

S6 Fig. Pseudo-gel image of gDNA samples sonicated to different fragment lengths from Agilent 2100 Bioanalyzer with a High Sensitivity DNA Chip (2100 Expert version B.02.10.SI764).

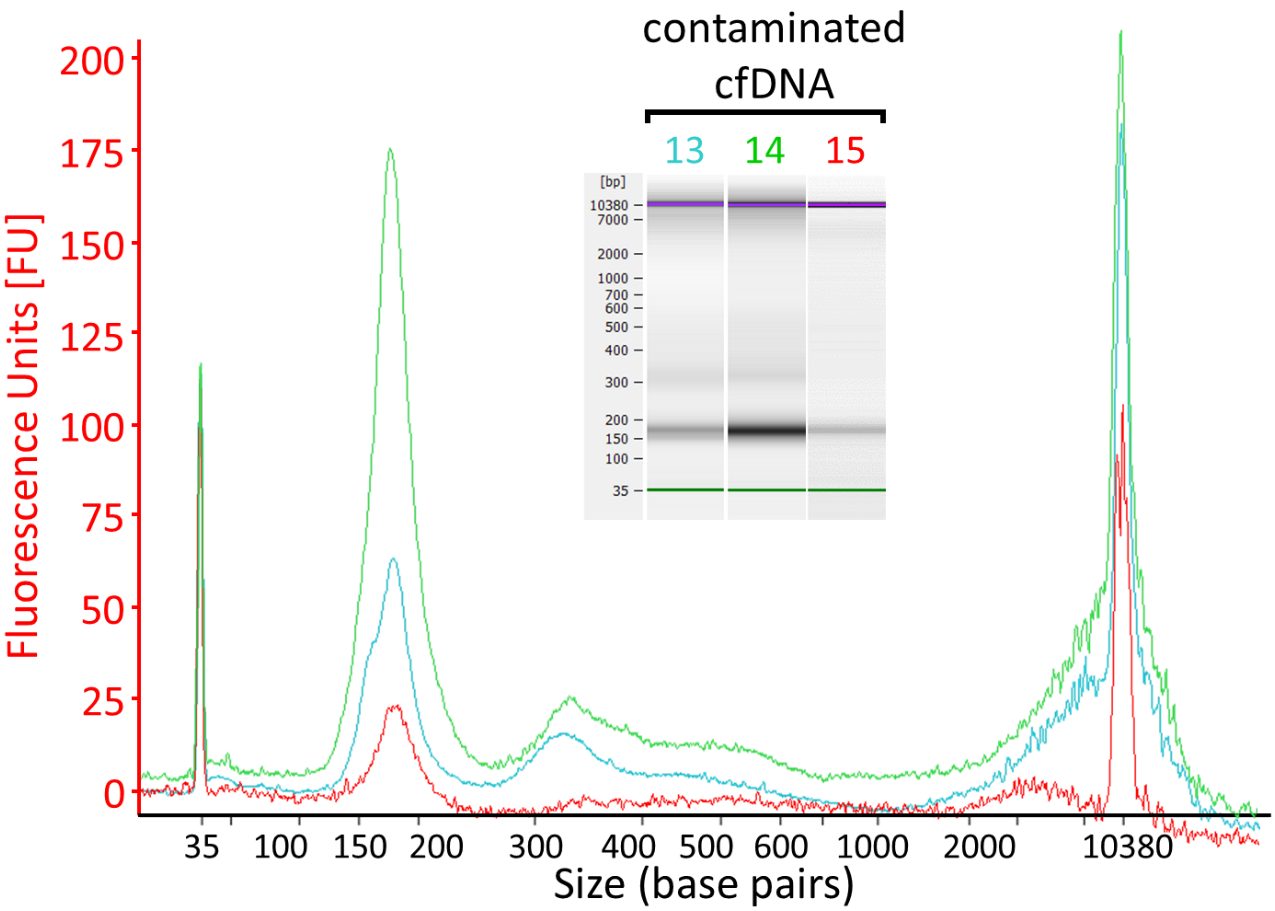

S7 Fig. Fragment size distributions of cfDNA samples excluded from analysis due to HMW DNA contamination. Two of these samples (13-14) were extracted using QIAamp Circulating Nucleic Acid Kit and one using in-house phenol-chloroform method (15). Results from Agilent 2100 Bioanalyzer with a High Sensitivity DNA Chip. Electropherograms and pseudo-gel images from 2100 Expert version B.02.10.SI764.
